# Transcriptome analysis of the differential effect of the NADPH oxidase gene *RbohB* in *Phaseolus vulgaris* roots following *Rhizobium tropici* and *Rhizophagus irregularis* inoculation

**DOI:** 10.1186/s12864-019-6162-7

**Published:** 2019-11-04

**Authors:** Citlali Fonseca-García, Alejandra E. Zayas, Jesús Montiel, Noreide Nava, Federico Sánchez, Carmen Quinto

**Affiliations:** 1 Departamento de Biología Molecular de Plantas, Instituto de Biotecnología, Universidad Nacional Autónoma de México, Avenida Universidad 2001, Colonia Chamilpa, Cuernavaca, Morelos 62210 Mexico; 2 Department of Molecular Biology and Genetics, Aarhus University, C 8000 Aarhus, Denmark

**Keywords:** Transcriptome, *Phaseolus vulgaris*, *Rboh*, Nodulation, Mycorrhization, Symbiosis

## Abstract

**Background:**

Reactive oxygen species (ROS) are generated by NADPH oxidases known as respiratory burst oxidase homologs (RBOHs) in plants. ROS regulate various cellular processes, including the mutualistic interactions between legumes and nitrogen-fixing bacteria or arbuscular mycorrhizal (AM) fungi. *Rboh* is a multigene family comprising nine members (*RbohA–I*) in common bean (*Phaseolus vulgaris*)*.* The RNA interference-mediated silencing of *RbohB* (*PvRbohB*-RNAi) in this species diminished its ROS production and greatly impaired nodulation. By contrast, the *PvRbohB*-RNAi transgenic roots showed early hyphal root colonization with enlarged fungal hypopodia; therefore, we proposed that *PvRbohB* positively regulates rhizobial infection (*Rhizobium tropici*) and inhibits AM colonization by *Rhizophagus irregularis* in *P. vulgaris.*

**Results:**

To corroborate this hypothesis, an RNA-Seq transcriptomic analysis was performed to identify the differentially expressed genes in the *PvRbohB*-RNAi roots inoculated with *Rhizobium tropici* or *Rhizophagus irregularis*. We found that, in the early stages, root nodule symbioses generated larger changes of the transcriptome than did AM symbioses in *P. vulgaris*. Genes related to ROS homeostasis and cell wall flexibility were markedly upregulated in the early stages of rhizobial colonization, but not during AM colonization. Compared with AM colonization, the rhizobia induced the expression of a greater number of genes encoding enzymes involved in the metabolism of auxins, cytokinins, and ethylene, which were typically repressed in the *PvRbohB*-RNAi roots.

**Conclusions:**

Our research provides substantial insights into the genetic interaction networks in the early stages of rhizobia and AM symbioses with *P. vulgaris*, as well as the differential roles that *RbohB* plays in processes related to ROS scavenging, cell wall remodeling, and phytohormone homeostasis during nodulation and mycorrhization in this legume.

## Background

Phosphorus and nitrogen are essential elements, the deficiency of which restricts plant growth. The acquisition of these nutrients can be facilitated through symbiotic associations between leguminous roots and soil microorganisms [1]. Arbuscular mycorrhizal (AM) fungi associate with plant roots and mobilize phosphate and nitrogen from the soil to the plant partner. In legumes, gram-negative soil bacteria called rhizobia induce the formation of root nodules, specialized organs in which atmospheric dinitrogen is fixed by the bacterial microsymbiont into a form usable by plants [2].

In plant–AM associations, strigolactones exuded by the plant roots promote hyphal branching and the biosynthesis of lipochito-oligosaccharides called *Myc* factors [3], which induce several physiological and molecular responses in the plant partner. The mutual recognition of both the macro- and micro-symbionts leads to the plant-orchestrated formation of the fungal hypopodium on the surface of the root epidermis and the prepenetration apparatus in the underlying epidermal cell, which forms the entry route of the microsymbiont. At this stage, the hyphae grow and ramify both intra- and intercellularly in the root, entering the inner cortical cells to form branched structures called arbuscules. These arbuscules are surrounded by a plant-derived membrane, named the periarbuscular membrane, through which nutrients are exchanged between the fungus and the plant host [4].

The legume–rhizobia symbiosis is also initiated by a molecular dialogue between symbionts. The flavonoids secreted into the rhizosphere by the legume roots are perceived by the rhizobial bacteria, which respond by biosynthesizing and secreting lipochito-oligosaccharides known as Nod factors into the rhizosphere. These molecules are specifically recognized by the plant root hair cell receptors and induce several physiological, cellular, and molecular responses. First, the bacteria attach to the root hair tips, prompting the swelling and curling that entraps the microsymbiont within a so-called infection chamber. At this point, a tubular structure known as the infection thread (IT) is formed in the infection chamber, allowing the bacteria to enter the root hair cells. The IT migrates to the inner layers of the root cortex, which reestablish their mitotic activity to form the nodule primordium, the precursor of the nitrogen-fixing nodule. Finally, the bacteria are released from the IT into specific cells of the inner cortex, where they become bacteroids that transform atmospheric dinitrogen into ammonia, a source of nitrogen that is assimilable by the plant [5].

Both mutualistic associations originate in the rhizosphere, and although obvious differences exist between these two processes, several molecular signals are recruited by the plant cell for both symbioses. Cell imaging experiments using a calcium chameleon reporter revealed that rhizobial and AM symbionts both trigger calcium spiking in the root cells of *Medicago truncatula*, which is likely required for the formation of the pre-IT and the prepenetration apparatus, respectively [6]. The activation of calcium spiking in the epidermal cells requires the enzyme *3-HYDROXY-3-METHYLGLUTARYL CoA REDUCTASE1*, a key regulator of the mevalonate pathway reported to interact with the plasma membrane receptor-like kinase *SYMBIOSIS RECEPTOR KINASE/DOES NOT MAKE INFECTIONS2* (*SYMRK/DMI2*) [7–9]. Downstream, the calcium oscillations are decoded by a nuclear Ca^2+^/calmodulin-dependent protein kinase and its interaction partner, CYCLOPS [10, 11]. Nucleoporins and cationic channels located in the nuclear envelope are also part of the common symbiotic pathways [12]. Silencing or mutating these shared genes affects the initial stages of both AM and rhizobial symbioses.

Despite these similarities, reactive oxygen species (ROS) produced by NADPH oxidases in legumes, known as respiratory burst oxidase homologs (RBOHs), seem to play contrasting roles in these mutualistic relationships. In rhizobial symbiosis, the RBOHs promote nodulation; for example, silencing *RbohB* expression impairs IT progression and nodule development in *Phaseolus vulgaris* roots inoculated with *Rhizobium tropici*, while its overexpression enhances rhizobial infection [13, 14]. An analogous phenotypic effect was observed in *M. truncatula* root hairs inoculated with *Sinorhizobium meliloti* when *MtROP9* (encoding RHO-LIKE PROTEIN9, a Rho-like GTPase believed to positively regulate the RBOHs) was silenced [15]. Other stages of the nodulation process are also positively regulated by legume NADPH oxidases [16]; for instance, the downregulation of *MtRbohA* and *PvRbohB* expression significantly reduced nitrogen fixation in *M. truncatula* and *P. vulgaris* nodules, respectively [13, 17]. In AM symbiosis, however, mounting evidence suggests that RBOH-dependent ROS production must be switched off to facilitate the colonization process. Hyphal colonization is promoted in transgenic *MtROP9-*silenced roots [15], while the loss of function of *PvRbohB* enhances the size of the fungal hypopodium and promotes hyphal colonization in *P. vulgaris* composite plants [18]. The opposite effect was observed in *P. vulgaris* roots overexpressing *PvRbohB*, in which AM invasion was substantially reduced [14]. The *RbohE* promoter is active in the arbuscule-hosting cells of *M. truncatula*, while its silencing through RNA interference (RNAi) impairs arbuscule formation with multiple cell penetration attempts [19]. These reports further demonstrate the crucial and contrasting roles of RBOH-dependent ROS production in these mutualistic associations; however, recent work suggests that the role of RBOHs in legume–AM symbioses is more complex.

Studies of *PvRbohB-*silenced or *PvRbohB*-overexpressing transgenic bean roots revealed contrasting effects during the early stages of AM and rhizobial symbiotic processes in *P. vulgaris* [13, 14, 18], leading us to propose that RBOH-produced ROS perform differential functions during the initial stages of these two symbiotic processes. Here, we performed a transcriptomic analysis of *P. vulgaris* using RNA-Seq, with the aim of identifying genes that are differentially expressed between control and *PvRbohB*-silenced transgenic *P. vulgaris* roots inoculated with either *R. tropici* or *Rhizophagus irregularis*. This study unveils the transcriptomic profile of several biological processes in response to rhizobia inoculation, which is absent or only partially activated in the AM-inoculated roots.

## Results

### Transcriptomic sequencing of rhizobia and AM symbioses in *P. vulgaris*

Previous studies conducted in our laboratory showed that *PvRbohB* plays crucial and putatively contrasting roles in rhizobial and AM symbioses in *P. vulgaris* roots. Furthermore, under nonsymbiotic conditions, the lateral root densities of the transgenic *PvRbohB*-silenced (*PvRbohB*-RNAi) plants were shown to be reduced relative to the control, indicating that *PvRbohB* participates in *P. vulgaris* root development [13, 18]. To further explore the impact of *PvRbohB* silencing on *P. vulgaris* gene expression, the transcriptomes of the control (nonsilenced transgenic roots) and *PvRbohB-*RNAi roots inoculated with rhizobia (*R. tropici*) or AM fungi (*R. irregularis*) were analyzed using RNA-Seq. In each biological condition, more than 34 million reads were obtained. The read lengths were 75 to 101 bp, with an average quality score of 28 to 35 (Additional file 1: Table S1). Mapping the reads to the *P. vulgaris* reference genome revealed a 95 to 98% coverage of the approximately 23,000 unigenes for each condition (Additional file 2: Table S2). The data were deposited in the NCBI databases under the BioProject accession number PRJNA482464.

In order to evaluate the variability between biological replicates, we performed ordination analyses for control and *PvRbohB*-RNAi samples. The multidimensional scaling (MDS) analyses showed that the data sets displayed separated clustering by the control and *PvRbohB*-RNAi samples without inoculation and inoculated with rhizobia (Additional file 4: Figure S1). However, the clustering of the AM data showed that replica number three of the controls (Ctrl_Myc_3) and *PvRbohB*-RNAi (Bi_Myc_3) were outside of the ordering. This result was corroborated by a correlation analysis, where Pearson’s correlation coefficients between replicas were low when replicate number three was analyzed (Additional file 4: Figure S1). Although Pearson’s correlation coefficients of some uninoculated and rhizobia inoculated samples were not relatively high, the clustering was ordinated separately between the both mentioned conditions. Considering this variability of the AM data, we decided to delete the replica number three of the controls and *PvRbohB*-RNAi for further analysis.

### Comparative analysis of the transcriptomic profiles of rhizobia and AM symbioses in *P. vulgaris* at 7 days postinoculation

**Fig. 1:**
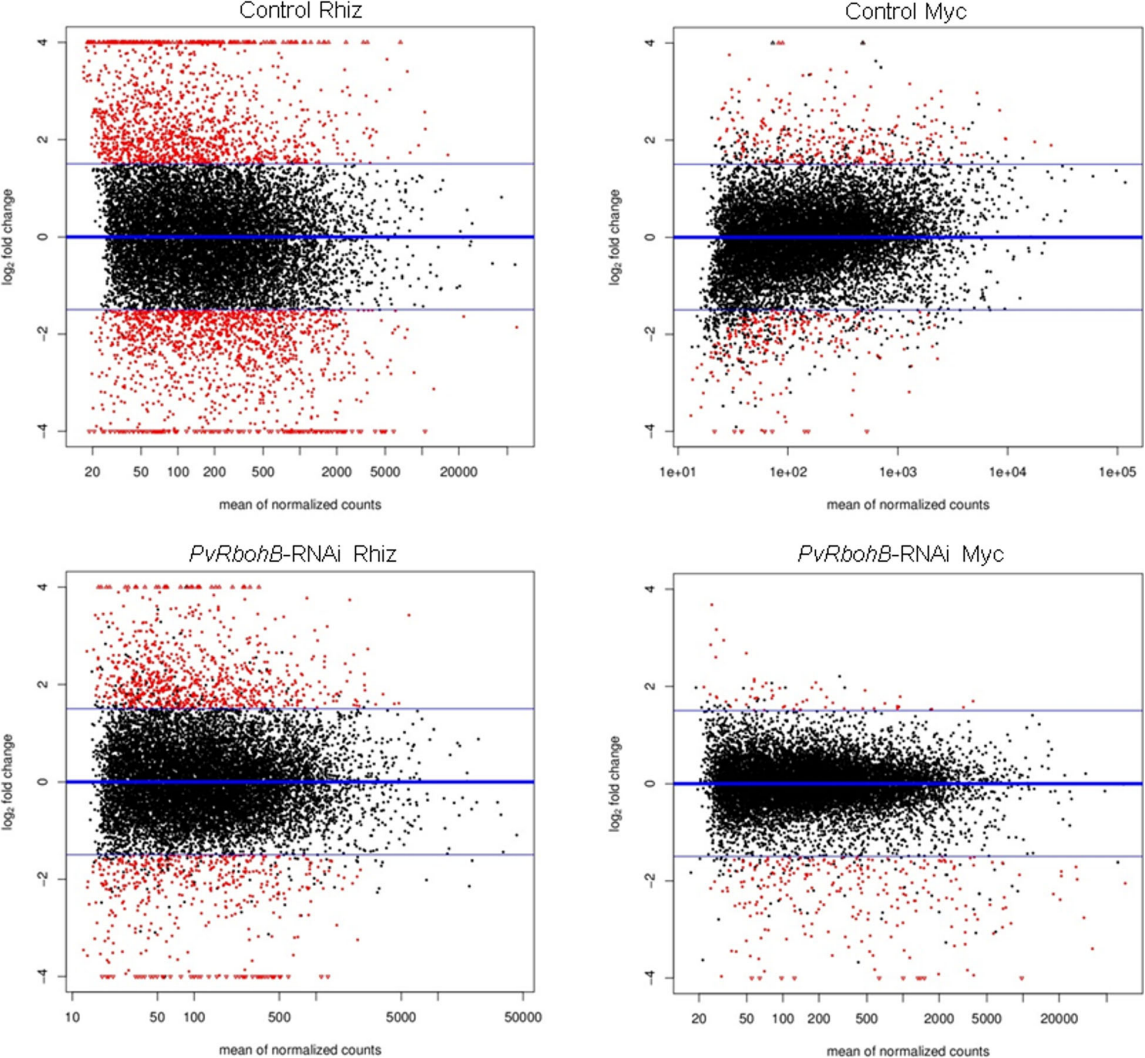
MAPlots of the transcriptomes of control and *PvRbohB*-RNAi *P. vulgaris* roots under nodulation and mycorrhization. Each plot shows the distribution of the Log2FoldChange values against the average of the normalized counts. Red dots are significantly differentially expressed genes, with a Log2FC ≥ 1.5 and P-adj/FDR ≤ 0.05. Rhiz, inoculated with *R. tropici*; Myc, inoculated with *R. irregularis*

**Fig. 2:**
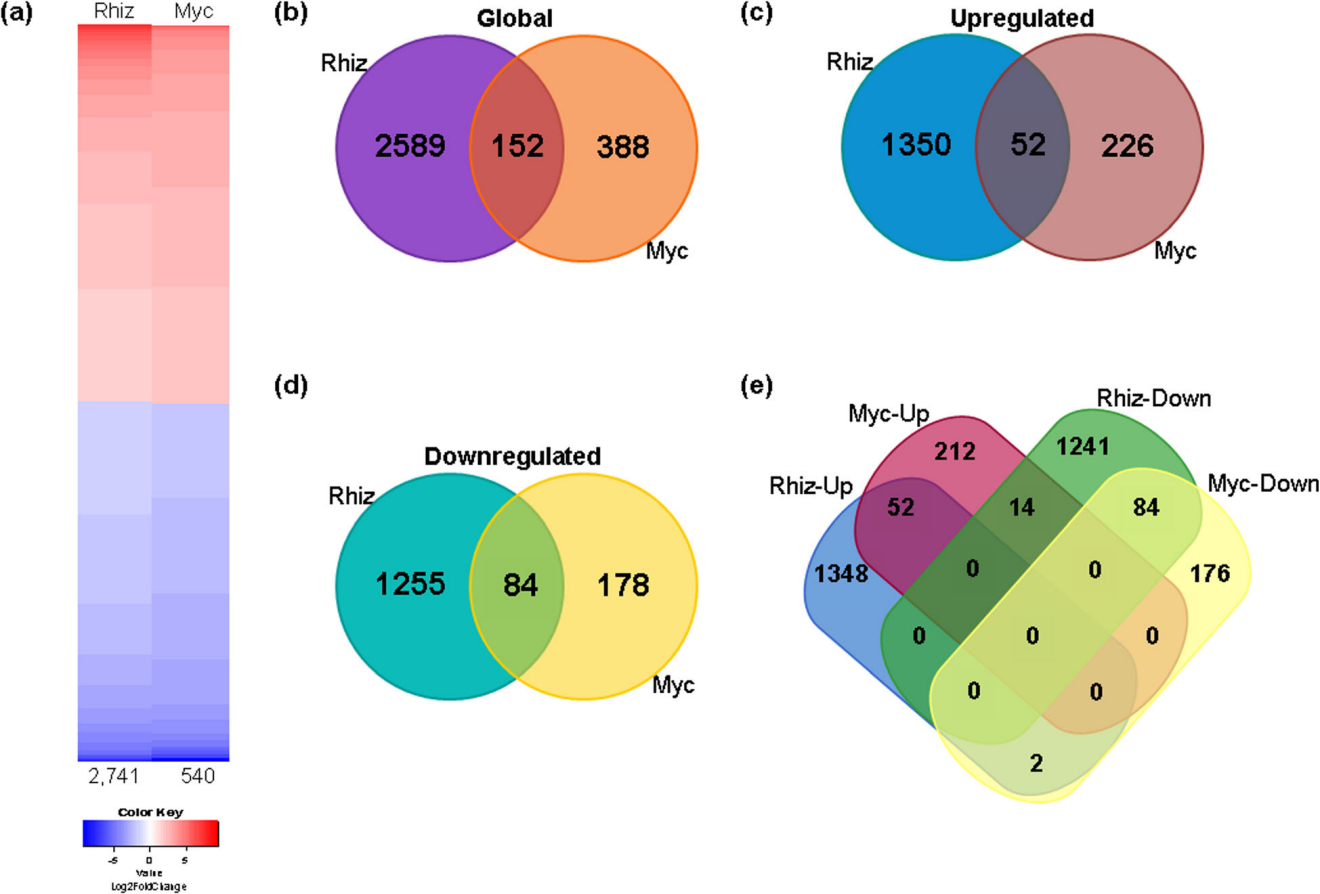
Global analysis of DEGs in rhizobia-inoculated and mycorrhized roots of *P. vulgaris*. **a** Heatmap of the total number of DEGs in roots at 7 dpi with *R. tropici* (Rhiz) or *R. irregularis* (Myc) relative to the noninoculated roots. **b-e** Venn diagrams indicate the total number of DEGs (**b**), and the numbers of upregulated (Up) (**c**), downregulated (Down) (**d**), and overlapping (**e**) genes in the rhizobia-inoculated and mycorrhizal roots. The DEGs were identified using a cutoff threshold of Log2FC ≥ 1.5 and a P-adj/FDR ≤ 0.05 in the DESeq, EdgeR, and NOISeq packages of Bioconductor R

Over the past decade, a compendium of transcriptomic resources has been developed for several legumes in rhizobial and AM symbioses [20–27]; nevertheless, the early stages of rhizobia- and AM-inoculated roots have rarely been explored and compared. Here, we found that, at 7 days postinoculation (dpi), 2741 genes were differentially expressed in roots inoculated with rhizobia relative to the uninoculated control (Figs. 1 and 2), using a cutoff threshold of ≥1.5 Log2FoldChange and a FDR-adjusted *P*-value of ≤0.05. However, only 540 genes were differentially expressed between AM- and uninoculated roots (Figs. 1 and 2). The proportion of upregulated and downregulated genes was similar in rhizobial- and AM-inoculated *P. vulgaris* roots (Fig. 2a), though only 152 genes were shared (Fig. 2b). A total of 1402 and 278 were upregulated differentially expressed genes (DEGs) in the rhizobial and AM roots, respectively, of which 52 upregulated DEGs were shared between both datasets (Fig. 2c). The rhizobia-inoculated roots had 1339 downregulated genes, while the AM roots had only 262 downregulated genes, 84 of which were shared between both biological treatments (Fig. 2d). Only 16 genes were found to be differentially regulated in the two symbioses; two were upregulated during nodulation and downregulated in mycorrhization, while 14 genes were downregulated in nodulation and upregulated during mycorrhization (Fig. 2e). These results suggest that these genes could play important differential roles in the early stages of nodulation and mycorrhization; however, further functional analyses are required to test this hypothesis.

The DEGs were annotated functionally within three Gene Ontology (GO) categories: biological process (BP), molecular function (MF), and cellular component (CC) (Additional file 5: Figure S2). There were clear differences in upregulated and downregulated genes between nodulation and mycorrhization conditions in BP. Amongst the upregulated genes, the response to stress, biosynthetic process, small molecule metabolic process, and cellular protein modification process constitute approximately 60% of the GO terms for nodulation condition. These same categories were less represented in the upregulated GO terms under mycorrhization conditions; catabolic process and cellular nitrogen compound metabolic process constituted around 40% of the GO terms under mycorrhization. Regarding the MF category, ion binding was the most abundant group in both biological treatments. Particularly in mycorrhized roots, the GO terms of up- and downregulated genes presented a similar composition, with a slight induction of genes related to kinase activity. However, in nodulated roots, several functional categories were downregulated, highlighting ion binding and oxidoreductase activity. In the CC category, both the up- and downregulated genes under mycorrhization conditions constituted three main groups: nucleus, endoplasmic reticulum, and plasma membrane. By contrast, under nodulation conditions, up- and downregulated genes had different functional groups, sharing only protein containing complex. Thus, GO term analysis revealed that the vast genetic reprograming observed in the early stages of nodulation and the more moderate changes observed during early mycorrhization largely involved genes associated with biological processes and cellular components (Additional file 5: Figure S2).

### Effect of *PvRbohB* silencing on the rhizobia transcriptome and AM symbioses in *P. vulgaris* at early stages of colonization

**Fig. 3:**
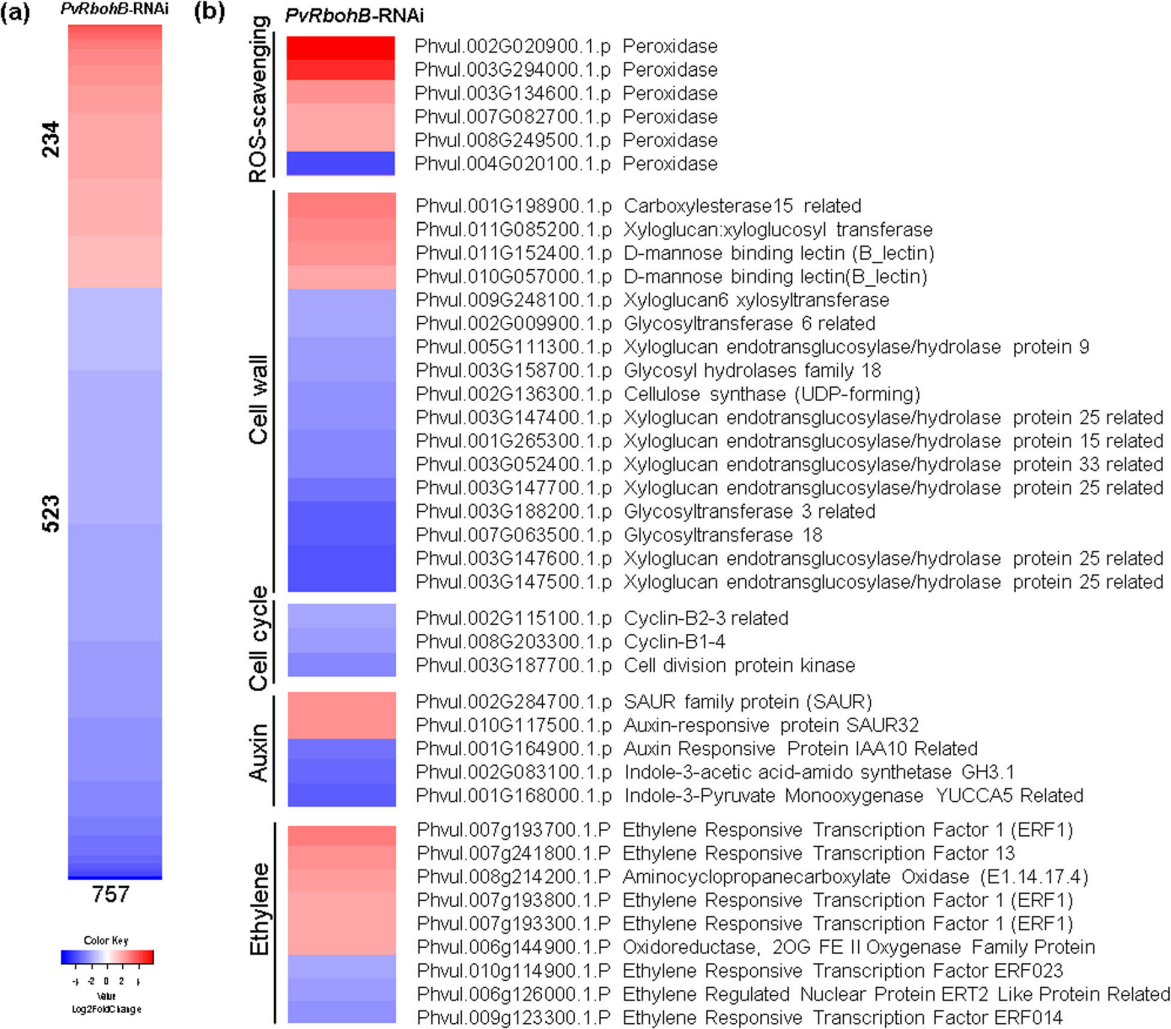
Global analysis of the DEGs in *PvRbohB*-RNAi *P. vulgaris* roots under nonsymbiotic conditions. **a** Heatmap of all DEGs. **b** Heatmap analyses of ROS-scavenging, cell wall, and cell cycle genes. The color bars represent the Log2FoldChange of the DEGs, with red and blue representing the upregulated and downregulated genes, respectively. A cutoff threshold of Log2FC ≥ 1.5 and P-adj/FDR ≤ 0.05 was used

*PvRbohB* silencing is known to affect the expression of several genes involved in nodulation and mycorrhization in *P. vulgaris* [13, 18]; however, this gene is also expressed in several organs under nonsymbiotic conditions, and its silencing negatively affects the development of the lateral roots [28]. In this study, we found that *PvRbohB* silencing causes differential expression of 757 genes in noninoculated *P. vulgaris* roots, of which 234 were upregulated and 523 were downregulated (Fig. 3a). This result shows that *PvRbohB* upregulates a greater number of genes than it downregulates. Several peroxidases and ethylene-related genes were induced in the *PvRbohB*-RNAi roots, suggesting a possible increase in the ROS and ethylene levels of these plants. ROS, which are known to be involved in a variety of processes in plants, could potentially be upregulated by ethylene [29]. *PvRbohB* silencing repressed the expression of genes involved in cell wall remodeling, such as *CELLULOSE SYNTHASE* and *XYLOGLUCAN ENDOTRANSGLUCOSYLASE/HYDROLASE*, together with important genes in the cell cycle and auxin biosynthesis, such as the gene encoding *THE INDOLE-3-PYRUVATE MONOOXYGENASE* YUCCA5 (Fig. 3b). Furthermore, a global functional annotation of the DEGs using GO terms indicated an induction in the expression of genes involved in biological regulation and catabolic processes, those with transferase and transmembrane transferase activities, as well as those involved in extracellular processes (Additional file 6: Figure S3). By contrast, the silencing of *PvRbohB* repressed the expression of genes related to signal transduction, cellular nitrogen compound metabolic processes, and kinase activity (Additional file 6: Figure S3), suggesting that *PvRbohB* plays a role in the signaling and gene regulation processes of *P. vulgaris*.

**Fig. 4:**
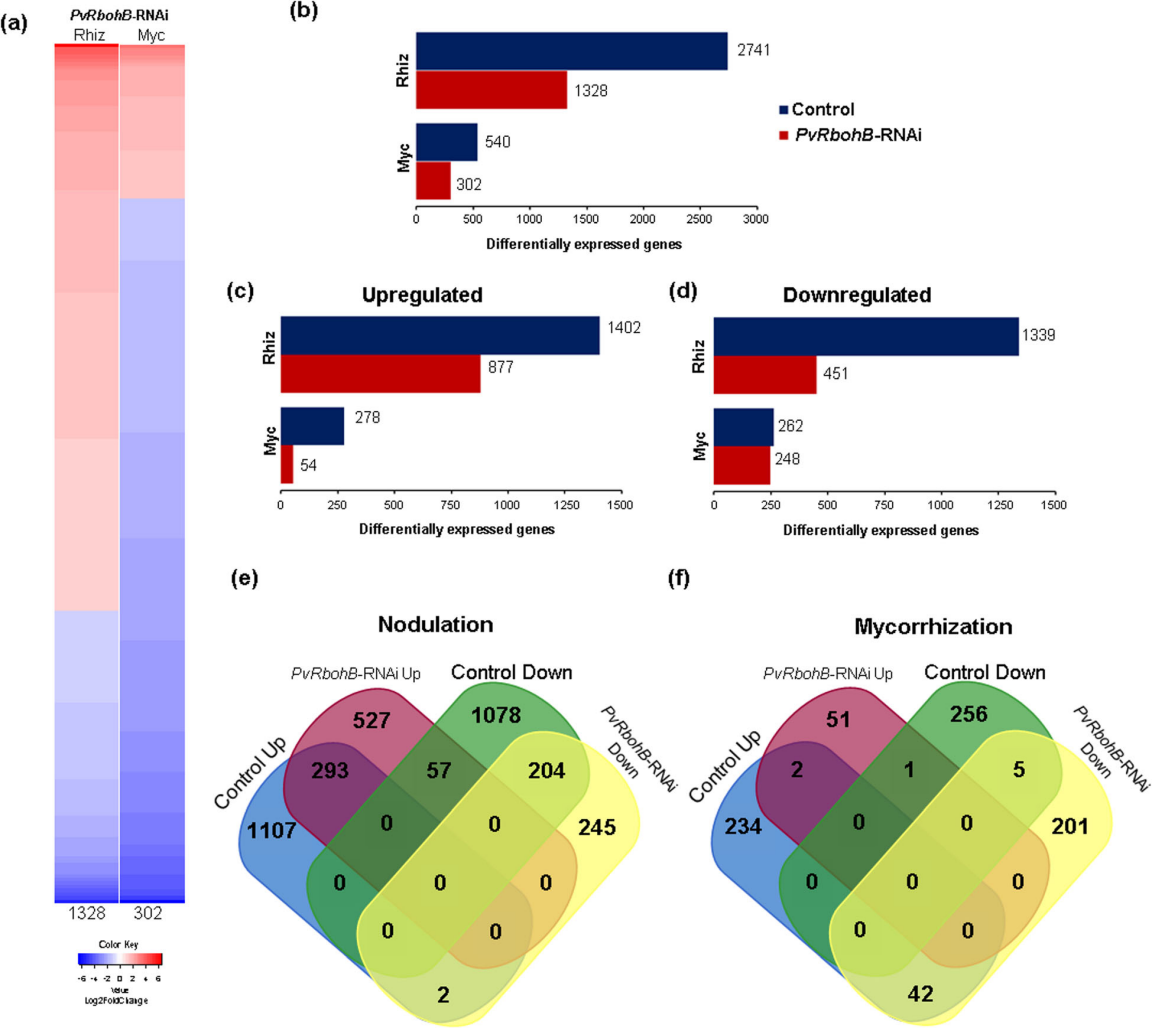
Global analysis of the DEGs in rhizobia-inoculated and mycorrhized *PvRbohB*-RNAi roots. **a** Heatmap of all DEGs between *PvRbohB*-RNAi roots inoculated with *R. tropici* (Rhiz) or *R. irregularis* (Myc) compared to the noninoculated *PvRbohB*-RNAi roots. Number of total DEGs (**b**), upregulated DEGs (Up) (**c**), and downregulated DEGs (Down) (**d**) identified between the control and *PvRbohB*-RNAi roots under nodulation and mycorrhization conditions. Venn diagrams show the intersections between the upregulated and downregulated DEGs shared between the nodulation (**e**) and mycorrhization (**f**) processes in control and silenced roots. A cutoff threshold of Log2FC ≥ 1.5 and P-adj/FDR ≤ 0.05 was used

The inoculation of *PvRbohB*-RNAi roots with *R. tropici* or *R. irregularis* affected the expression of 1328 and 302 genes, respectively (Fig. 4a–b). In response to rhizobial inoculation, 1402 genes were upregulated in the control roots; however, only 293 of these genes were also induced in the inoculated *PvRbohB*-RNAi roots (Fig. 4c, e). Similarly, in mycorrhized roots, of the 278 genes upregulated in the control transgenic roots, only two were induced in *PvRbohB*-RNAi roots (Fig. 4c, f). Furthermore, 42 of the genes upregulated during mycorrhization in the control roots were downregulated in the *PvRbohB*-RNAi roots (Fig. 4f).

The normal transcriptional repression of a large set of genes during both symbiotic processes was substantially altered in the *PvRbohB*-RNAi roots. Approximately 85% of the downregulated genes in the rhizobial-inoculated control roots were not downregulated in the *PvRbohB-*silenced roots at 7 dpi (Fig. 4d, e). Furthermore, only 2% of the 262 downregulated genes in the mycorrhized control roots were similarly downregulated in the *PvRbohB*-RNAi roots, while an additional 201 genes were downregulated in these transgenic plants, suggesting that the early stages of AM symbiosis were strongly impacted by *PvRbohB* silencing. Under nodulation conditions, 57 of the upregulated genes in the *PvRbohB*-RNAi roots were downregulated in the control roots. Moreover, the functional annotation of these genes indicated that the main changes caused by the silencing of *PvRbohB* at the functional level were also observed in *P. vulgaris* under nodulation conditions, while there were specific modifications to catabolic processes, signal transduction, transmembrane transporter activity, and plasma membrane at 7 dpi with AM (Additional file 7: Figure S4). These results could be related to the early stages of colonization by both microsymbionts.

To assess the efficacy and specificity of the *RbohB* gene silencing, we quantified *PvRbohB* expression using both RNA-Seq and reverse-transcription quantitative PCR (RT-qPCR) data (Additional file 8: Figure S5). The *PvRbohB*-RNAi roots were found to have an 80% reduction in the transcript level of this gene relative to the control, supporting the resulting phenotype. Therefore, the RT-qPCR results support the findings obtained in the RNA-Seq analysis.

### Regulation of ROS- and cell wall-related genes in *PvRbohB*-RNAi roots under symbiotic conditions

**Fig. 5:**
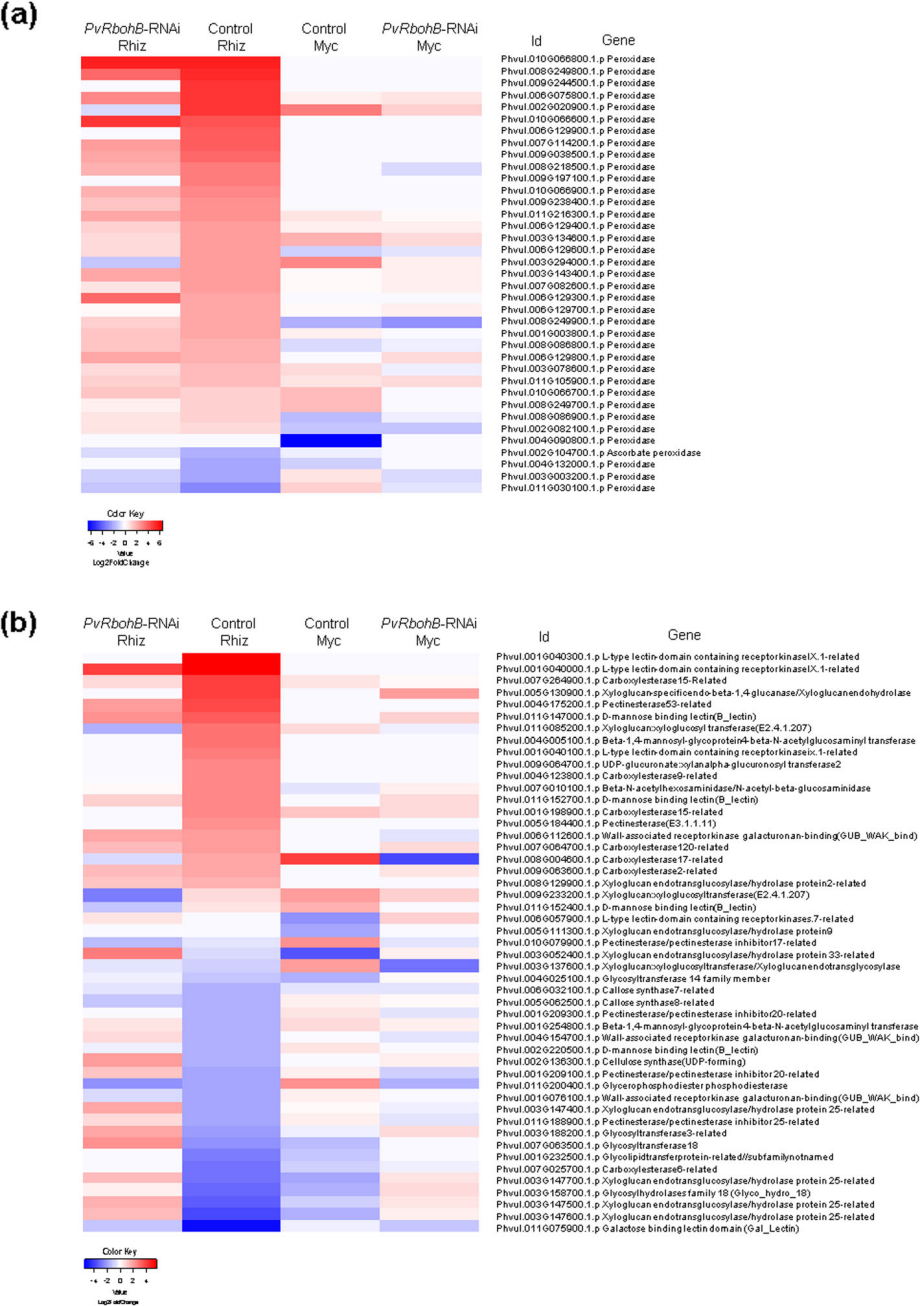
Heatmap of the expression patterns of genes related to ROS scavenging and cell wall biosynthesis. DEGs encoding proteins related to ROS metabolism (**a**) and cell wall biosynthesis (**b**) in the control and *PvRbohB*-RNAi *P. vulgaris* roots, at 7 dpi with rhizobia or AM. The color bars represent the Log2FoldChange of the DEGs, with red and blue representing the upregulated and downregulated genes, respectively. A cutoff threshold of Log2FC ≥ 1.5 and P-adj/FDR ≤ 0.05 was used

As previously mentioned, *PvRbohB* silencing negatively impacts nodulation and positively affects mycorrhization in *P. vulgaris*. We evaluated the effect of *PvRbohB-*RNAi on the expression of the ROS-scavenging genes, since RBOHs are prominent ROS-generating systems in plants [30]. In this study, we found that the expression levels of 28 ROS-scavenging genes were increased in the control roots inoculated with rhizobia, most of which encoded class-III peroxidases (Fig. 5a). In the *PvRbohB*-silenced roots, however, only 12 peroxidase genes were upregulated. In mycorrhized control roots, only five ROS-scavenging peroxidase genes were upregulated and three were downregulated; however, the expression levels of these genes were unaffected in the *PvRbohB*-RNAi roots (Fig. 5a).

ROS metabolism is tightly linked to cell wall remodeling. Hydroxyl radicals are involved in the loosening of cell walls via an apoplastic peroxidase-dependent mechanism, and hydrogen peroxide is involved in cell wall lignification [31, 32]. The cell wall must be dynamically modulated to allow for the successful invasion of rhizobia and AM [33]. Here, we identified DEGs related to plant cell wall biogenesis, such as those encoding BETA-1,4-GLUCANASES, PECTINESTERASE, LECTINS, CARBOXYLESTERASE, and UDP-GLUCURONATE:XYLANALPHA GLUCURONOSYLTRANSFERASE, between the inoculated and noninoculated plants. Rhizobial inoculation induced the differential expression of more genes than did AM colonization (Fig. 5b), reflecting the differences in the developmental programs and kinetics of these symbioses. Under nodulation conditions, genes involved in cell wall biogenesis were induced in the *PvRbohB*-RNAi roots; however, some of these were downregulated in the control roots. Similarly, *PvRbohB* silencing had a clear influence on cell wall biosynthesis genes during mycorrhization, as only those encoding XYLOGLUCAN:XYLOGLUCOSYLTRANSFERASE and CARBOXYLESTERASE shared the same expression profile as the mycorrhized control roots (Fig. 5b). These results confirm the role of *RbohB* in growth and development processes in *P. vulgaris*.

### Signaling of phytohormone-related genes during rhizobial and AM symbioses

**Fig. 6:**
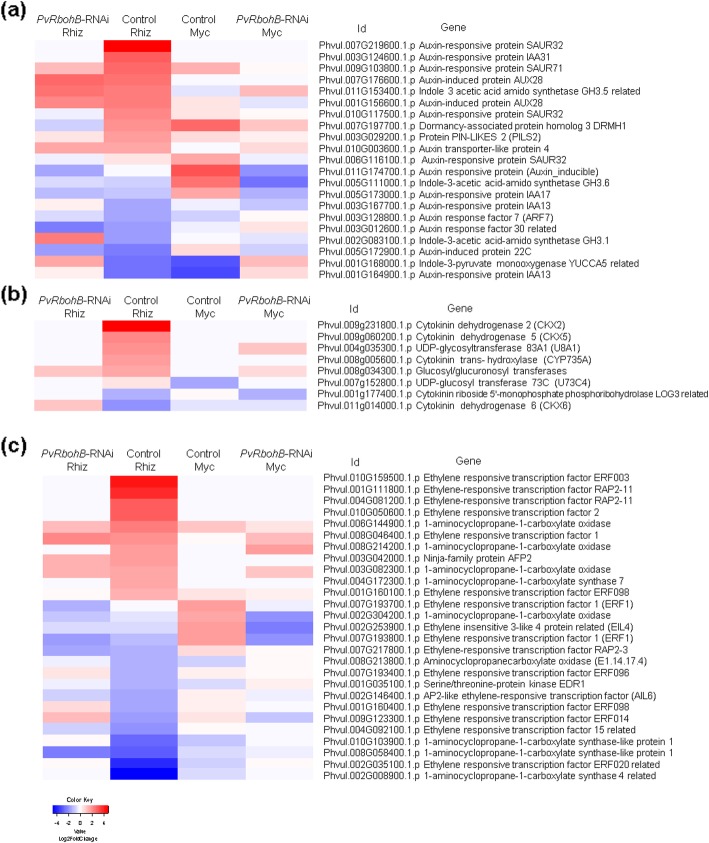
Heatmap of the expression patterns of genes involved in phytohormone metabolism. DEGs related to the auxin (**a**), cytokinin (**b**), and ethylene (**c**) signaling pathways in the control and *PvRbohB*-RNAi *P. vulgaris* roots, at 7 dpi with rhizobia or AM. The color bars represent the Log2FoldChange of the DEGs, with red and blue representing the upregulated and downregulated genes, respectively. A cutoff threshold of Log2FC ≥ 1.5 and P-adj/FDR ≤ 0.05 was used

Numerous reports have demonstrated a close relationship between ROS and several phytohormones during plant development and responses to stresses [34]. The production of ROS by AtRBOHD is an important signal for auxin-regulated cell division during lateral root formation in *Arabidopsis thaliana* [35]. We identified 15 upregulated and 6 downregulated auxin-related genes in the rhizobia-inoculated control roots, which were mainly early auxin response and regulatory genes (Fig. 6a). The five upregulated early auxin signaling genes in the mycorrhized control roots differed from the auxin-induced genes in the nodulated control roots, indicating a differential regulation of the auxin-related genes in these symbiotic processes (Fig. 6a). These results were confirmed in KEGG plant hormone signal transduction where genes related to synthesis and regulation of the auxin pathway were induced in rhizobia-inoculated and mycorrhized control roots, but also downregulated in rhizobia-inoculated control roots (Additional file 9: Figure S6, Additional file 10: Figure S7, Additional files 11: Figure S8 and Additional file 12: Figure S9). However, in the *PvRbohB*-RNAi roots, the auxin pathway genes were induced in rhizobia-inoculated roots and downregulated in mycorrhized control roots (Additional file 13: Figure S10, Additional file 14: Figure S11, Additional files 15: Figure S12 and Additional file 16: Figure S13).

Cytokinins are key phytohormones that play a variety of roles in nodulation [36]; however, they are not known to make a major contribution to AM symbiosis [37]. In the control *P. vulgaris* roots inoculated with rhizobia, five genes related to the biosynthesis, activation, and degradation of cytokinins were upregulated relative to the inoculated control; however, none of these genes were induced in the *PvRbohB*-silenced roots (Fig. 6b). In the mycorrhized control roots, the induction of genes related to cytokinin signaling was not observed, although two such genes were repressed. By contrast, two genes (*UDP-GLYCOSYLTRANSFERASE 83A1* and *GLUCOSYL/GLUCURONOSYL TRANSFERASE*) involved in cytokinin metabolism were upregulated in the *PvRbohB*-RNAi roots during mycorrhization (Fig. 6b). These results demonstrate the distinct roles played by cytokinins in these symbioses.

Ethylene has been widely studied as a negative regulator of both rhizobial and mycorrhizal symbioses [38]. It negatively regulates the infection and organogenesis of determinate and indeterminate nodulating species [39–41]. In this analysis, we identified 11 upregulated and 13 downregulated genes related to the response and biosynthesis of ethylene in the control rhizobia-inoculated roots (Fig. 6c). A previous study of a pea (*Pisum sativum*) mutant line (E107) with altered symbiotic features and ethylene responses suggested that ethylene could be responsible for its decreased AM colonization [42]. Here, genes related to the response and biosynthesis of ethylene were also induced in control mycorrhized roots; however, none of them were downregulated. In the *PvRbohB*-RNAi roots, the genes involved in the biosynthesis and response of ethylene were not induced to the same extent as in the control roots, suggesting that less ethylene production and a smaller ethylene response takes place in the *PvRbohB*-silenced roots during rhizobial symbioses (Fig. 6c). Similarly, genes involved in the ethylene response pathway were downregulated or not induced in *PvRbohB*-RNAi roots inoculated with AM (Fig. 6c). These results suggest that *PvRbohB* plays a crucial role in the ethylene responses of *P. vulgaris* under both symbiotic processes.

### Validation of DEG expression by RT-qPCR

**Fig. 7:**
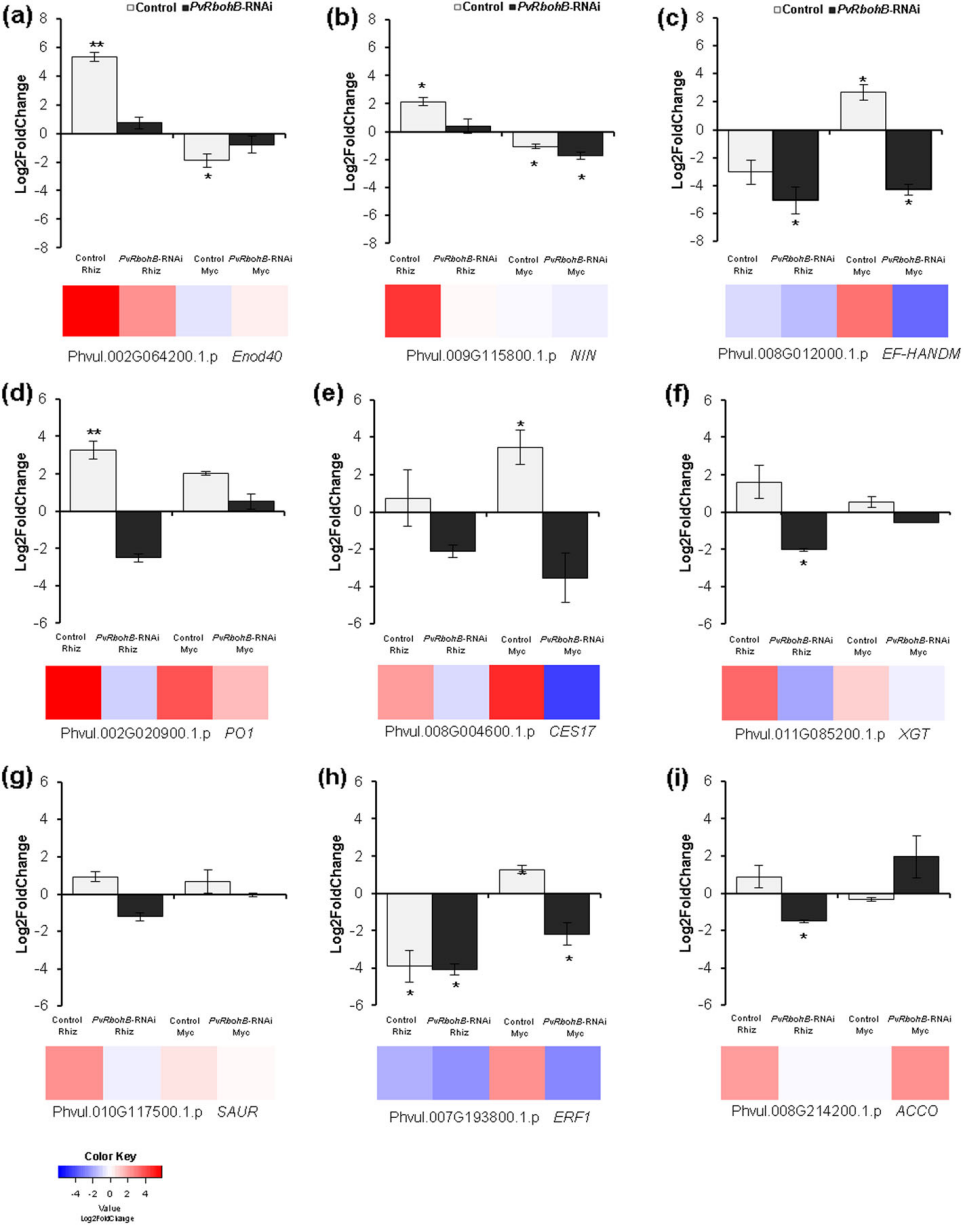
Validation of the expression patterns of genes induced in control and *PvRbohB*-RNAi roots. The expression levels of *PvENOD40* (**a**), *PvNIN* (**b**), *PvEF-HANDM* (**c**), *PvPO1* (**d**), *PvCES17* (**e**), *PvXGT* (**f**), *PvSAUR* (**g**), *PvERF1* (**h**), and *PvACCO* (**i**) were evaluated using RT-qPCR in *P. vulgaris* transgenic roots at 7 dpi with AM or rhizobia. The heatmaps of the expression of the corresponding genes, obtained using RNA-Seq, are shown below the graphs (**a–i**). The RT-qPCR data were normalized to the expression of *PvEF1α*. The RT-qPCR bars are the means ± SE of at least two independent biological replicates, with three technical repeats (*n* > 10). The asterisks represent statistically significant differences between the noninoculated and inoculated samples, as revealed using an unpaired Student’s *t*-test (*P < 0.05; **P < 0.01). The color bars of the heatmaps represent the Log2FoldChange, where red and blue represent the upregulated and downregulated genes, respectively

To validate the transcriptome results, we determined the transcript abundance of 9 DEGs in the control and *PvRbohB*-RNAi roots under nodulation and mycorrhization conditions using RT-qPCR. As molecular markers of the nodulation process, the transcript levels of the early nodulins *EARLY NODULIN40* (*ENOD40*) and *NODULE INCEPTION* (*NIN*) were examined (Fig. 7a–b). These genes are upregulated during the formation of the IT and nodule primordia [43, 44]. For mycorrhization, we evaluated one upregulated gene identified in the RNA-Seq analysis, *EF-HAND CALCIUM-BINDING DOMAIN-CONTAINING PROTEIN* (*EF-HANDM*) (Fig. 7c). The results obtained using RT-qPCR and RNA-Seq were highly consistent (Fig. 7). The *PvENOD40* and *PvNIN* transcript levels were significantly increased in the control rhizobia-inoculated roots (Fig. 7a–b); however, these nodulins were not induced in the *PvRbohB*-RNAi roots, supporting our previous findings [13, 18]. In the control roots colonized by AM, the *PvEF-HANDM* transcripts were significantly upregulated but were remarkably downregulated in the *PvRbohB*-RNAi roots (Fig. 7c).

Furthermore, under nodulation conditions, *PEROXIDASE1* (*PvPO1*), *CARBOXYLESTERASE17* (*PvCES17*), *XYLOGLUCAN:XYLOGLUCOSYLTRANSFERASE* (*PvXGT*), *PvSAUR*, and *AMINOCYCLOPROPANE CARBOXYLATE OXIDASE* (*PvACCO*) were upregulated in the control roots and downregulated in the *PvRbohB*-RNAi roots (Fig. 7d, f, g, i). Similarly, *PvCES17*, *PvXGT*, *PvSAUR*, *PvACCO*, and *ETHYLENE RESPONSIVE TRANSCRIPTION FACTOR1* (*PvERF1*) were upregulated in the mycorrhized control roots but downregulated in the mycorrhized *PvRbohB*-silenced roots (Fig. 7e–i). These results showed a strong correlation with the transcriptome data (Additional file 17: Figure S14), supporting our analysis of the involvement of *PvRbohB* in regulating ROS-scavenging, cell wall, and phytohormone-related genes.

## Discussion

### Root nodule symbiosis triggers a vast transcriptional response, while AM symbiosis has a moderate response in *P. vulgaris* transgenic roots at the early stages of colonization

Plants promote associations with a wide variety of microorganisms that result in beneficial interactions to both partners. The most studied symbiotic interactions are those between legumes and nitrogen-fixing rhizobial bacteria and between plants and AM with different objectives and strategies [45]. Numerous investigations based on gene expression profiles have aimed to decipher the functional mechanisms that rhizobial and AM symbioses establish with legumes [20–27]. However, the early stages of rhizobia-inoculated and AM roots have not been studied as much. In this work, we analyzed and compared the transcriptomic profile of rhizobial-inoculated and mycorrhized roots during the early stages of both symbioses (7 dpi). Our data show that rhizobial symbiosis triggers a vast transcriptional response in the first 7 days after inoculation while AM symbiosis has a more moderate response (Fig. 2); however, the opposite scenario was previously observed at the later stages of symbiosis, when *P. vulgaris* roots had 3219 DEGs at 14 dpi but only 2645 DEGs under nodulation conditions [25]. In bird’s foot trefoil (*Lotus japonicus*) roots, previously generated transcriptomic data indicated that 3248 DEGs were present at 12 dpi with *Mesorhizobium loti*, while 3641 DEGs were detected in AM roots at 27 dpi, of which only 275 overlapped with the rhizobial-symbiosis DEGs [23]. The low number of genes with differential expression patterns between nodulation and mycorrhization at 7 dpi contrasts with the numerous DEGs previously detected at 14 dpi [25]. The lag in the kinetics of these symbiotic processes could partially explain this difference. Additionally, root nodule symbiosis involves the biogenesis of a nitrogen-fixing organ, while only a few cortical cell divisions have been observed in response to AM in wild-type roots [5, 46]. Furthermore, the most prominent functional categories assigned to upregulated genes in rhizobia-inoculated roots were, in contrast, assigned to the downregulated genes in the mycorrhized roots, suggesting a differential effect for these biological processes (Additional file 6: Figure S3).

### *PvRbohB* silencing causes transcriptomic changes in *P. vulgaris* with and without symbionts

RBOHs, plant NADPH oxidases, are one of the specific enzymes that produce ROS in plants. An increasing body of evidence indicates that ROS production by RBOHs is involved in the regulation of root hair and primary root growth [47]. Loss-of-function studies of several RBOHs resulted in a reduction in primary root growth in Arabidopsis [48, 49]. Particularly, *PvRbohB* silencing showed a marginal reduction in the growth of the main root and a significant reduction in lateral root density [13]. In this work, the expression levels of the cell wall-remodeling genes, as well as genes involved in the cell cycle and the biosynthesis of and response to auxins, were considerably downregulated in the *PvRbohB-*silenced roots (Fig. 2b). These results are consistent with the 15% reduction in lateral root density, as well as the differences in lateral root lengths detected in *PvRbohB*-RNAi roots at 1 to 7 days postemergence [13, 28], confirming that *PvRbohB* is an important component of growth and development in *P. vulgaris* roots.

Under symbiotic conditions, *PvRbohB* silencing affected the expression of approximately half the number of DEGs in the nonsilenced roots under nodulation and mycorrhization conditions. In rhizobial-inoculated roots, 20% of the induced genes in control roots were upregulated in *PvRbohB*-RNAi roots, while in mycorrhized roots, only 0.7% exhibited a similar response in *PvRbohB*-RNAi roots. These results suggest that *PvRbohB* silencing had a more drastic impact on the transcriptome changing of the initial steps of AM. Consistent with these results, a significant repression of gene expression was observed during the initial stages of rhizobial infection and AM colonization in *M. truncatula* transgenic roots with silenced *MtROP9i* relative to the control [50]. By contrast, a transcriptome analysis performed on the roots of *M. truncatula* inoculated with *S. meliloti* revealed that the expression of 67% of the genes required for the symbiosis were affected by a treatment with diphenylene iodonium, an inhibitor of ROS production [51]. Furthermore, several genes involved in cell wall remodeling, early nodulins, and biosynthesis of and response to phytohormones such as auxins and ethylene had an opposite expression pattern between rhizobial-inoculated and mycorrhized control roots against *PvRbohB*-RNAi inoculated roots. Likewise, transcription factors such as WRKYs which can function up- and downstream of hormones and control developmental processes via auxins, cytokinins, and brassinosteroids [52]. Our data strongly suggest that *PvRbohB* plays a central role in cell wall remodeling and the response to phytohormones under symbiotic conditions.

### Cell wall remodeling affected by *PvRbohB*-RNAi roots during rhizobial and AM symbioses

ROS are oxygen products that play dual roles in plants, both as toxic compounds and as key regulators of many biological processes [53]. ROS produced by RBOHs have shown to have specific effects in legume–rhizobia and mycorrhizal symbioses [13, 14, 17, 18, 50]. Particularly, studies of *PvRbohB-*silenced transgenic bean roots revealed a positive impact during the early stages of AM and a negative impact in rhizobial symbiotic processes in *P. vulgaris* [13, 14, 18]. In addition, ROS have been detected during IT growth and the formation of nodule primordia in alfalfa (*Medicago sativa*) [54]. Recently, it was observed that the suppression of the immune system by a calcium-dependent protein kinase-Rboh complex is required for rhizobial colonization in *M. truncatula* [55]. ROS production is a component of the early nodulation signaling pathway initiated by the perception of Nod factors [16, 40, 56–58]. This process is accompanied by the expression of class-III peroxidases [56, 59, 60], which have two catalytic activities (peroxidative and hydrolytic) that enable them to either generate ROS (**˙**OH and HOO**˙**) or decrease ROS levels by sequestration of hydrogen peroxide during polymerization of the cell wall [61]. In this work, we observed that *PvRbohB* silencing affects the expression of a large number of peroxidases in rhizobia-inoculated and mycorrhized roots (Fig. 5a), indicating that *PvRbohB* plays a crucial role during ROS-mediated signaling in both symbiotic processes. In addition, genes involved in cell wall biogenesis presented a differential expression in control and *PvRbohB*-silenced transgenic roots under both symbiotic processes (Fig. 5b). In rhizobial-inoculated roots, *PvRbohB* silencing induced some cell wall biogenesis genes that were downregulated in the control roots, such as those encoding GLYCOSYLTRANSFERASE, CELLULOSE SYNTHASE, and BETA-1,4-MANNOSYL-GLYCOPROTEIN 4-BETA-*N*-ACETYLGLUCOSAMINYL TRANSFERASE. The last of these proteins is one of the most important enzymes involved in the regulation of glycoprotein oligosaccharide biosynthesis. It catalyzes the addition of *N*-acetylglucosamine in a beta 1–4 linkage to the beta-linked mannose of the trimannosyl core of the N-linked sugar chains, altering not only the composition, but also the conformation, processing, and elongation of the N-glycan molecule [62, 63]. The effect of *PvRbohB* silencing on the transcriptional response of this battery of genes supports the defects observed in the nodulation program of the *RbohB*-RNAi roots [13]. Several *Rboh* members are known to be important players in the developmental programs of different tissues and organs in a variety of plant species [47–49, 64].

### *PvRbohB* silencing affects phytohormone-related genes under symbiotic conditions

The plant hormone auxin is crucial to all aspects of plant growth and development. In legumes, auxin biosynthesis and signaling occur during the formation of the IT [65]. Auxin-responsive and signaling components, such as *SMALL AUXIN UP RNA1* (*SAUR1*), *GRETCHEN HAGEN3.1* (*GH3.1*), *AUXIN RESPONSE FACTOR 16a* (*ARF16a*), and *INDOLE-3-ACETIC ACID INDUCIBLE9* (*IAA9*), were induced in *M. truncatula* root hairs at 1 to 5 dpi with *S. meliloti* [39, 66]. Here, we also observed the induction of the early auxin response genes, such as *SAUR32/71*, *GH3.5*, and *IAA28*, in the rhizobia-inoculated roots; however, *ARF7* and *GH3.1/3.6* were repressed, suggesting a differential role for these genes in *P. vulgaris*. Approximately 45% of the auxin-related genes observed in the rhizobia-inoculated control roots presented different expression patterns in the *PvRbohB*-RNAi roots, including *SAUR32*, *IAA13*, and *DRMH1* (Fig. 6a). However, the role of auxins in mycorrhizal symbioses is not clear, as biochemical and molecular studies have revealed variable levels of auxins and auxin-related molecules throughout these processes [37, 67–69]. Studies using auxin mutants have suggested that auxin perception is required for arbuscule development and the establishment of mycorrhizal symbiosis, at least for modulating strigolactone levels during this process [70]. Similar to our observations in the rhizobia-inoculated roots, the early auxin response genes, such as *IAA17*, *GH3.6*, and *SAUR32/71*, were induced in the mycorrhized control roots, while *ARF7* was repressed (Fig. 6a). The auxin-regulated protein GH3 and other transcripts encoding putative auxin response factors were induced during the colonization of rice (*Oryza sativa*) and *M. truncatula* roots by *Rhizoglomus irregulare* [71, 72]; however, in contrast to our data, an *ARF* (*ARF18*) transcript was previously reported to be induced in sunflower (*Helianthus annuus*) roots during colonization by *R. irregulare* [73]. *PvRbohB*-RNAi abolished the differential expression levels of these genes in both the root nodule and AM symbioses, although genes such as *GH3.1* were induced in the rhizobia-inoculated roots (Fig. 6a). Similarly, homologs of the auxin transporter AUX1-like genes were found to be slightly induced by rhizobial infection in *P. vulgaris*, but not in *PvRbohA*-RNAi roots [74]. These data suggest that *PvRbohB* has an important function in the regulation and response to auxin in both microbial symbioses.

The cytokinins are essential for nodule organogenesis, a process that is orchestrated by the CYTOKININ DEHYDROGENASES (CKXs) in *L. japonicus* [75–77]. In this study, *PvCKX2* and *PvCKX5* were induced in the rhizobia-inoculated control roots, confirming the role of the cytokinins at the early stages of nodulation in *P. vulgaris* (Fig. 6b). These genes were downregulated in the *PvRbohB*-silenced roots, however, in which only *CKX6* and *GLUCOSYL/GLUCURONOSYL TRANSFERASE* were upregulated, suggesting a crucial role for *PvRbohB* in regulating cytokinin biosynthesis during nodulation in *P. vulgaris*. Nevertheless, a studies involving the *CYTOKININ RESPONSE1* (*CRE1*) cytokinin receptor mutant in *M. truncatula* have suggested that the cytokinins may be less critical for AM colonization [37]. Our data generally support this conclusion, but also show that *PvRbohB* plays a role in the biosynthesis of cytokinins during *P. vulgaris*–AM symbiosis. This difference between symbioses may be partially due to the central role that cytokinins play in the orchestration of cell division during nodulation, which is not required for mycorrhizal colonization. This nodulation-related mitosis was affected in the *PvRbohB*-RNAi transgenic roots, which showed a drastic reduction in mitotic activity compared to the infected control roots [13], which are not required for mycorrhizal colonization.

Cytokinin signaling displays broad interactions with other hormone signaling pathways, such as ethylene. Cytokinin can stimulate the production of ethylene [78], which is a negative regulator of both rhizobial and mycorrhizal symbioses [38]. In this work, genes involved in the biosynthesis and response of ethylene presented differential expression in control and *PvRbohA*-RNAi roots (Fig. 6c). Particularly, the homologs of two key ethylene biosynthesis regulators, *1-AMINOCYCLOPROPANE-1-CARBOXYLATE (ACC) SYNTHASE* and *ACC OXIDASE*, were upregulated in the control roots, confirming previous reports of an increase in ethylene production during the early stages of nodulation in *P. vulgaris* [41, 79–81]. Similarly, in mycorrhized roots, the homologs of *ACC OXIDASE* were upregulated under control and *PvRbohB* silencing conditions. By contrast, the expression levels of several other homologs of these genes were repressed at the same time point, indicating a complex regulation of ethylene metabolism in this legume. Our results confirm the central role of auxins, cytokinins, and ethylene in the nodulation and mycorrhization processes in *P. vulgaris* and suggest that *PvRbohB* could be an important player in the homeostasis of these phytohormones in both microbial symbioses.

## Conclusions

In this article, we present a comparative transcriptome analysis of the *P. vulgaris* control and *PvRbohB*-RNAi roots inoculated with rhizobia and AM fungi. Our results indicate that root nodule symbiosis induce considerable transcriptomic changes, with the AM fungi inducing less of a response than rhizobia during the early stages of their respective *P. vulgaris* symbioses. Additionally, a compendium of DEGs related to ROS scavenging, cell wall remodeling, and phytohormone homeostasis during nodulation and mycorrhization in *P. vulgaris* were found to be affected by *PvRbohB* silencing. These results provide important information on the symbiotic gene signaling networks involved in the early stages of rhizobial and mycorrhizal colonization as well as the differential effects of a NADPH oxidase gene, *RbohB*, on these processes.

## Methods

### Plant materials, microsymbiont inoculation, and growth conditions

Mesoamerican *P. vulgaris* cv. Negro Jamapa was obtained from the local market. Transgenic *PvRboh*-RNAi and nonsilenced (control) roots were generated in a transformation using *Agrobacterium rhizogenes* K599 under laboratory conditions according to Instituto de Biotecnología-UNAM guidelines [13]. The composite plants were transferred into pots and inoculated with the respective microsymbionts. The roots were inoculated with *Rhizophagus irregularis* (800 spores per plant; INI-FAP biofertilizer, produced at Campo Experimental Rosario Izapa, Chiapas, Mexico) or *Rhizobium tropici* (strain CIAT899; 1 ml per seedling at OD_600_ = 0.05) with the same conditions used previously [13, 18]. The plants inoculated with *R. irregularis* were irrigated twice a week with half-strength B&D solution [82] containing a low concentration of potassium phosphate (10 μM K_2_HPO_4_) to facilitate AM colonization [83]. To promote nodulation, the composite plants inoculated with rhizobia were watered with B&D medium without nitrate (KNO_3_), while the noninoculated plants (*n* = 10 per biological replicate) were irrigated with B&D solution supplemented with 8 mM potassium nitrate to prevent nodulation. At 7 dpi, the roots (*n* = 5–10 per condition) were harvested for RNA isolation, immersed in liquid nitrogen, and stored at − 80 °C until use. Before freezing, a section of each transgenic root was analyzed under the microscope to confirm fungal colonization (*R. irregularis*) or bacterial infection (*R. tropici*). Noreide Nava undertook the formal identification of the plant material used in this work under our laboratory conditions.

### RNA extraction, library preparation, and sequencing

For the transcriptome profiling, high-quality total RNA was isolated from the frozen tissues of three biological replicates with 30–5 plants using a ZR Plant RNA MiniPrep kit following the manufacturer’s instructions (Zymo Research, Irvine, CA, USA). Any contamination from genomic DNA was eliminated by incubating the samples with RNAase-free DNAase (1 U μl^− 1^) at 37 °C for 15 min and at 65 °C for 10 min. The RNA integrity numbers (RINs) [84] and the concentrations of the RNA samples were determined using an Agilent 2100 Bioanalyzer platform (Agilent Technologies, Santa Clara, CA, USA) with a Nano 6000 kit (Thermo Fisher Scientific, Waltham, MA, USA). Only samples with RIN values of 8.0 or above were used for the RNA-Seq analysis. cDNA libraries were individually generated for each sample using the TruSeq RNA-Seq Sample Prep kit (Illumina, San Diego, CA, USA), following the manufacturer’s protocol. Libraries were sequenced as 75- to 100-bp paired-end reads using an Illumina HiSeq 2000.

### RT-qPCR assays

To validate the RNA-Seq results, an RT-qPCR analysis was performed for a selection of highly upregulated or downregulated genes. High-quality total RNA was isolated from the frozen root tissues using a ZR Plant RNA MiniPrep kit (Zymo Research), following the manufacturer’s instructions. The integrity of the RNA was confirmed using gel electrophoresis, and the RNA concentration was determined with a NanoDrop2000 spectrophotometer (Thermo Fisher Scientific, Waltham, MA, USA). Any genomic DNA was removed using RNAase-free DNAase (1 U μl^− 1^; Roche, Basel, Switzerland), following the manufacturer’s recommendations. The RT-qPCR analysis was performed using a Maxima SYBR Green/Fluorescein qPCR kit (Thermo Fisher Scientific), according to the following instructions: each reaction contained 7.5 μl Maxima SYBR Green/Fluorescein qPCR Master Mix (2X) (Thermo Scientific, USA), 0.5 μl cDNA samples, and 0.45 μl of each primer (10 μM) in a reaction system of 15 μl. The thermal cycle was as follows: 95 °C for 10 min, 40 cycles of 95 °C for 15 s, 60 °C for 30 s, and 72 °C for 30 s. To corroborate the absence of contaminant DNA, a control sample without reverse transcriptase was included. The relative expression values were determined using the formula 2^–CT^, where the cycle threshold value CT is equal to the Ct of the gene of interest minus the Ct of the reference gene, *P. vulgaris EF1α* [85]. At least two biological replicates were performed, and each dataset was generated using three technical repeats. The gene-specific oligonucleotides used for the RT-qPCR analysis are listed in Additional file 3: Table S3.

### Alignment and data analysis

To estimate the transcript abundance in each condition, the sequence quality was analyzed using FastQC software (www.bioinformatics.babraham.ac.uk/projects). The high-quality paired-end reads were mapped onto the *P. vulgaris* G19833 genome, obtained from the National Center for Biotechnology Information (NCBI) Genome database (Assembly GCA_000499845.1; https://www.ncbi.nlm.nih.gov/genome/380?genome_assembly_id=48590), using the BWA software package for mapping low-divergence sequences ([86]; http://bio-bwa.sourceforge.net). We used the “bwa mem” command to align the reads with default parameters. For each sample, both paired ends were run together; only the samples from the transgenic *PvRboh*-RNAi roots inoculated with *R. tropici* were mapped as single-end reads. The aligned sequencing reads were counted using htseq-count, a Python script with default parameters that taked as input a Binary Alignment Map (BAM) file and a General Feature Format (GFF) file with gene models [87]. To evaluate the intrinsic variability within the data groups, a multidimensional scaling (MDS) ordination method was used, which graphically displays the dissimilarities between the samples. Also, the Pearson’s correlation coefficients between the replicates were estimated using the *cor* function of the ggpubr package and plotted with the function ggplot of the ggplot2 package all in R to confirm the clustering observed in the MDS plots. The results shown reflect at least two biological replicates for each treatment.

The DEGs were identified using the software packages DESeq [88], NOIseq, and edgeR [89], which offer an exact statistical test. A counts per million (CPM) of 5 and a log_2_ fold change (Log2FoldChange) ≥ 1.5 with a false discovery rate (FDR) or P-adjusted ≤0.05 was used as a cutoff for the identification of upregulated and downregulated genes. This analysis was subsequently summarized in a MAPlot, in which the significance of the differential expression results is compared to the Log2FoldChange values and the mean number of normalized counts per gene. These analyses were performed using *Integrated Differential Expression Analysis MultiEXperiment* [90].

Additionally, the DEGs were functionally annotated using the Blast2GO software ([88], https://www.blast2go.com/), supported by GO terms, InterPro domains, and enzyme codes. A GO term overview was performed using a GO-slim analysis implemented in Blast2GO software using the UniProt database [91]. The pathway annotations were based on the Kyoto Encyclopedia of Genes and Genomes (KEGG) pathway (www.genome.jp/kegg) [92]. The distribution and abundance (Log2FoldChange) of these genes were presented in Venn diagrams using the function *draw.quad.venn* in the VennDiagram package in R and in heatmaps with the function *heatmap.2* of the gplot package in R, respectively. An unpaired Student’s *t*-test was performed using the *t.test* function of the stats package in R.

## Supplementary information

**Additional file 1: Table S1.** Statistical quality control analysis of the raw sequence data, performed using FastQC software.

**Additional file 2: Table S2.** Statistics of the reads for each condition mapped onto the reference genome of P. vulgaris using the Burrows-Wheeler Aligner (BWA).

**Additional file 3: Table S3.** Genespecific oligonucleotides used in this study.

**Additional file 4: Figure S1.** Intrinsic variability within the data groups. Multidimensional Scaling plots (MDS) (**a**-**c**) and Pearson correlation (**d**-**f**) of the different replicas per condition of control and *PvRbohB*-RNAi samples of the transcriptome uninoculated (**a**, **d**), inoculated with rhizobia (**b**, **e**), and inoculated with AM (**c**, **f**). Ctrl: control roots; Bi: *PvRbohB*-RNAi; Rhiz: inoculated with *R. tropici*; Myc: inoculated with *R. irregularis*; 1: replicate 1; 2: replicate 2; 3: replicate 3.

**Additional file 2: Figure S2.** Functional annotation of the DEGs in rhizobia-inoculated (Rhiz) and mycorrhized (Myc) roots of *P. vulgaris*. The bars show the percentages of the upregulated (**a, c**, **e**) and downregulated (**b**, **d**, **f**) DEGs annotated with GO terms for the functional categories of biological processes (**a**–**b**), molecular functions (**c**–**d**), and cellular components (**e**–**f**). A cutoff threshold of Log2FC ≥ 1.5 and P-adj/FDR ≤ 0.05 was used, and GO terms were assigned using a GO-Slim analysis of the UniProt database in Blast2GO.

**Additional file 6: Figure S3.** Functional annotation of the DEGs in the *PvRbohB*-RNAi roots in nonsymbiotic conditions of *P. vulgaris*. Functional annotation of the upregulated and downregulated DEGs annotated with GO terms of the functional categories of biological processes (**a**), molecular functions (**b**), and cellular components (**c**). A cutoff threshold of Log2FC ≥ 1.5 and P-adj/FDR ≤ 0.05 was used, and GO terms were assigned using a GO-Slim analysis of the UniProt database in Blast2GO.

**Additional file 7: Figure S4.** Functional annotation of the DEGs in rhizobia-inoculated and mycorrhized *PvRbohB*-RNAi roots, relative to the control. The bars show the percentages of upregulated (**a, c**, **e**) and downregulated (**b**, **d**, **f**) DEGs annotated with GO terms of the functional categories of biological processes (**a**–**b**), molecular functions (**c**–**d**), and cellular components (**e**–**f**). A cutoff threshold of Log2FC ≥ 1.5 and P-adj/FDR ≤ 0.05 was used, and GO terms were assigned using a GO-Slim analysis against the UniProt database in Blast2GO.

**Additional file 8: Figure S5.** Transcript levels of *PvRbohB* in the control and *PvRbohB*-RNAi roots used in this study. The plot includes RNA-Seq data and validating RT-qPCR data. Bars indicate the means ± SE.

**Additional file 9: Figure S6.** KEGG pathway map of plant hormone signal transduction for upregulated genes in rhizobia-inoculated control roots.

**Additional file 10: Figure S7.** KEGG pathway map of plant hormone signal transduction for downregulated genes in rhizobia-inoculated control roots.

**Additional file 11: Figure S8.** KEGG pathway map of plant hormone signal transduction for upregulated genes in mycorrhized control roots.

**Additional file 12: Figure S9.** KEGG pathway map of plant hormone signal transduction for downregulated genes in mycorrhized control roots.

**Additional file 13: Figure S10.** KEGG pathway map of plant hormone signal transduction for upregulated genes in rhizobia-inoculated *PvRbohB*-silenced roots.

**Additional file 14: Figure S11.** KEGG pathway map of plant hormone signal transduction for downregulated genes in rhizobia-inoculated *PvRbohB*-silenced roots.

**Additional file 15: Figure S12.** KEGG pathway map of plant hormone signal transduction for upregulated genes in mycorrhized *PvRbohB*-silenced roots.

**Additional file 16: Figure S13.** KEGG pathway map of plant hormone signal transduction for downregulated genes in mycorrhized *PvRbohB*-silenced roots.

**Additional file 17: Figure S14.** Correlation of the RNA-seq data and RT-qPCR expression profiles. The mean transcriptional changes (Log2FoldChange) of ROS-scavenging, cell wall, and phytohormone-related genes between the rhizobial-inoculated (**a**) and AM (**b**) roots at 7 dpi.

## Data Availability

The data were deposited in the NCBI databases under the BioProject accession number PRJNA482464, the BioSample accession numbers SAMN09831769–SAMN09831794, and the Sequence Read Archive accession numbers SRR7693915–SRR7693917, SRR7696192–SRR7696194, SRR7696200–SRR7696202, SRR7696204–SRR7696206, SRR7696589–SRR7696591, and SRR7696208–SRR7696210.

## Abbreviations

AM: Arbuscular mycorrhizal
BAM: Binary alignment map
BP: Biological process
CC: Cellular component
CPM: Count per million
DEG: Differential expressed genes
FDR: False discovery rate
GO: Gene ontology
IT: Infection thread
KEGG: Kyoto Encyclopedia of Genes and Genomes
MDS: Multidimensional scaling
MF: Molecular function
NCBI: National Centre for Biotechnology Information
RBOH: Respiratory burst oxidase homologs
RNAi: RNA interference
ROS: Reactive oxygen species
RT-qPCR: Reverse-transcription quantitative of polymerase chain reaction
SAM: Sequence alignment map
SYMRK: Symbiosis receptor kinase/does not make infections 2
