# Supplementary material for: Transcriptome analysis of the differential effect of the NADPH oxidase gene RbohB in Phaseolus vulgaris roots following Rhizobium tropici and Rhizophagus irregularis inoculation
Source: BMC Genomics. 2019 Nov 4;20:800. doi: 10.1186/s12864-019-6162-7 (PMC6827182; doi:10.1186/s12864-019-6162-7)
Supplement: Supplementary file 1 — Additional file 1: Table S1. Statistical quality control analysis of the raw sequence data, performed using FastQC software. [file 12864_2019_6162_MOESM1_ESM.pdf]

Table S1. Statistical quality control analysis of the raw sequence data, performed using FastQC software.

	**Control**	*PvRbohB*-RNAi	**Control_Rhiz***	*PvRbohB*-RNAi**_Rhiz**	**Control_Myc***	*PvRbohB*-RNAi**_Myc**
Total number of raw reads	17,191,375x2	18,994,157x2	21,459,988x2	22,185,057x1	16,022,206x2	20,075,259x2
Total number of reads with poor quality	0	0	0	0	0	0
Reads length (bp)	101	101	75	75	101	101
GC%	45	44	47	47-48	45-46	44-46
Average quality score	37	38	35	35	37	38

*Rhiz, inoculated with *R. tropici*; Myc, inoculated with *R. irregularis*.
