# Supplementary material for: Transcriptome analysis of the differential effect of the NADPH oxidase gene RbohB in Phaseolus vulgaris roots following Rhizobium tropici and Rhizophagus irregularis inoculation
Source: BMC Genomics. 2019 Nov 4;20:800. doi: 10.1186/s12864-019-6162-7 (PMC6827182; doi:10.1186/s12864-019-6162-7)
Supplement: Supplementary file 2 — Additional file 2: Table S2. Statistics of the reads for each condition mapped onto the reference genome of P. vulgaris using the Burrows-Wheeler Aligner (BWA). [file 12864_2019_6162_MOESM2_ESM.pdf]

Table S2. Statistics of the reads for each condition mapped onto the reference genome of *P. vulgaris* using the Burrows-Wheeler Aligner (BWA).

| | **Control** | *PvRbohB*-RNAi | **Control_Rhiz*** | *PvRbohB*-RNAi**_Rhiz** | **Control_Myc*** | *PvRbohB*-RNAi**_Myc** |
|---|---|---|---|---|---|---|
| Percentage of mapped reads | 98.31–98.83 | 97.82–98.68 | 96.48–98.18 | 95.85–96.85 | 97.09–98.55 | 98.58–98.90 |
| Total number of mapped genes | 23,135 | 23,100 | 22,393 | 22,410 | 22,559 | 23,357 |

*Rhiz, inoculated with *R. tropici*; Myc, inoculated with *R. irregularis*.
