# Supplementary material for: Transcriptome analysis of the differential effect of the NADPH oxidase gene RbohB in Phaseolus vulgaris roots following Rhizobium tropici and Rhizophagus irregularis inoculation
Source: BMC Genomics. 2019 Nov 4;20:800. doi: 10.1186/s12864-019-6162-7 (PMC6827182; doi:10.1186/s12864-019-6162-7)
Supplement: Supplementary file 3 — Additional file 3: Table S3. Genespecific oligonucleotides used in this study. [file 12864_2019_6162_MOESM3_ESM.pdf]

Table S3. Gene-specific oligonucleotides used in this study.

Gene	Tag	Sequence of the primers	Amplicon size (bp)
PvEf1α	Ef1α-Up	GGT CAT TGG TCA TGT CGA CTC TGG	146
	Ef1α-Lw	GCA CCC AGG CAT ACT TGA ATG ACC	
PvENOD40	ENOD40q_F	AGT TTT GTT GGC AAG CAT CC	106
	ENOD40q_R	TAA GCA CAA GCA AAC TGT TG	
PvNIN	NIN-Fw	GGG GAT TCA GAG ATT TGC AG	101
	NIN-Rv	AAC CCA CTC TTG AGC ATC GT	
PvPR1	F96900	TTC CTC AAC GCT CAC AAC ACT GC	130
	R96900	CAG AGT GCA CCA TCT TGC AGT	
PvEF-HANDM	F12000	GGA AGC ATT CAG CGT ATC AGT ACG	139
	R12000	CTC CAC ATC AAT ACC GAA GTG GG	
PvPO1	F20900	GGT CAA CAA CTA TAG CAA GTG GCC	133
	R20900	TCA GTT CAG AGC CCT GCA GTT TG	
PvPO2	F34600	AAA GGG GCT TCT CAG TTC TGA CC	109
	R34600	CCC GAA GAA GAG GCC ACT ATC C	
PvSAUR	F17500	GGG AGA AGA GTC CCA GGA GTT TTC	100
	R17500	TGA CCA ACT TTG ATC GCC ATG CAC	
PvCDK20	F65900	CAG GCA AGG ATA CTT GTG GAG TCT	96
	R65900	AGC TTC ACG ATG TTG TTG GAG TGA C	
PvERF1	F93800	GTC AAC CAA TGA TGT TCT TGA CCC TC	114
	R93800	TCC ACC ACC AAT TGC TTT GCC TC	
PvCES17	F04600	ACA TGG CAC GGT GAA GTT GAG AG	125
	R04600	CCC TTTT CCC TGC TTT AGC CAA AG	
PvACCO	F14200	ATTGGACACTGTGGAGAGGTTGACT	121
	R14200	GTC CAT ATC CTT GAC CTC AGT TTG GAT G	
PvXGT	F85200	GGG GAC CTC AGC ATC AGA GAA	118
	R85200	ATT GCA GCA CCA AAG TAT CCA GAC TT	
