# Supplementary material for: Transcriptome analysis of the differential effect of the NADPH oxidase gene RbohB in Phaseolus vulgaris roots following Rhizobium tropici and Rhizophagus irregularis inoculation
Source: BMC Genomics. 2019 Nov 4;20:800. doi: 10.1186/s12864-019-6162-7 (PMC6827182; doi:10.1186/s12864-019-6162-7)
Supplement: Supplementary file 4 — Additional file 4: Figure S1. Intrinsic variability within the data groups. Multidimensional Scaling plots (MDS) (a-c) and Pearson correlation (d-f) of the different replicas per condition of control and PvRbohB-RNAi samples of the transcriptome uninoculated (a, d), inoculated with rhizobia (b, e), and inoculated with AM (c, f). Ctrl: control roots; Bi: PvRbohB-RNAi; Rhiz: inoculated with R. tropici; Myc: inoculated with R. irregularis; 1: replicate 1; 2: replicate 2; 3: replicate 3. [file 12864_2019_6162_MOESM4_ESM.pdf]

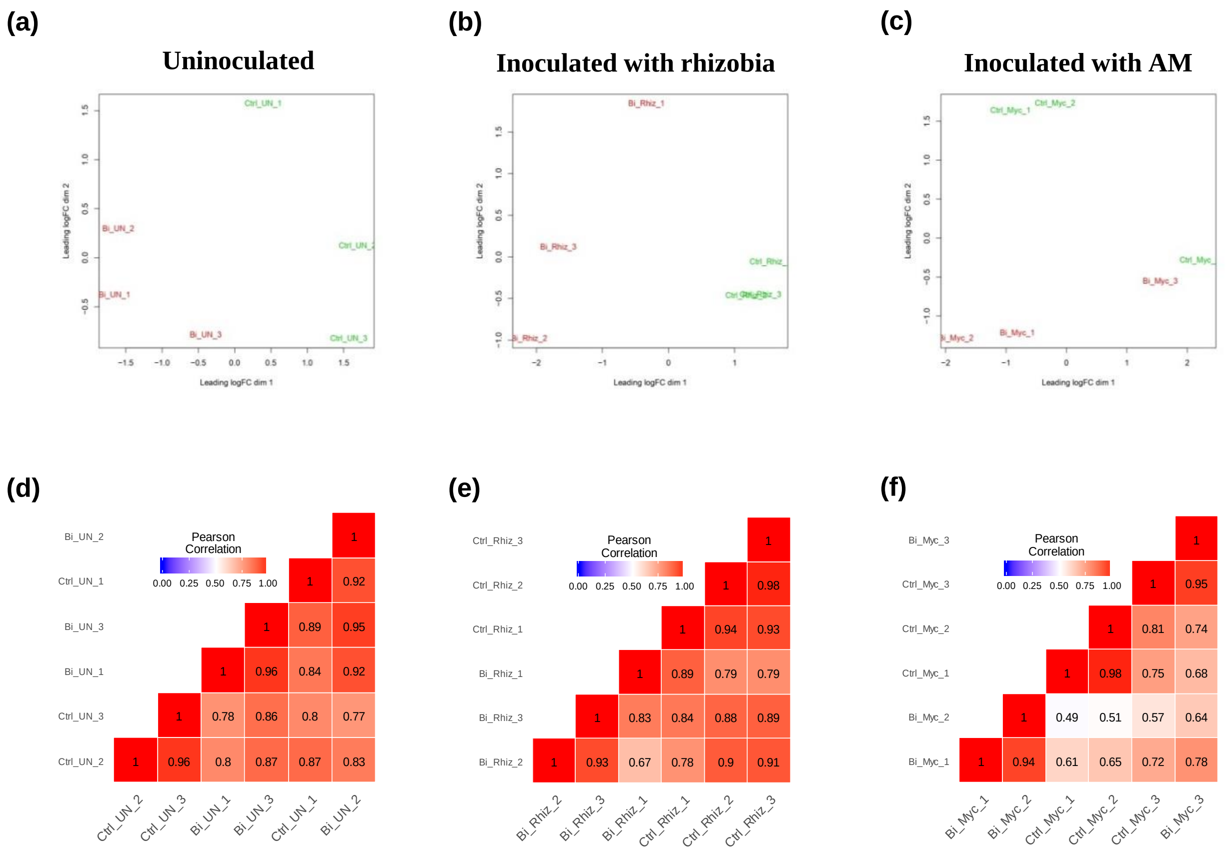

Figure S1 Intrinsic variability within the data groups. Multidimensional Scaling plots (MDS) (**a-c**) and Pearson correlation (**d-f**) of the different replicas per condition of control and *PvRbohB*-RNAi samples of the transcriptome uninoculated (**a**, **d**), inoculated with rhizobia (**b**, **e**), and inoculated with AM (**c**, **f**). Ctrl: control roots; Bi: *PvRbohB*-RNAi; Rhiz: inoculated with *R. tropici*; Myc: inoculated with *R. irregularis*; 1: replicate 1; 2: replicate 2; 3: replicate 3.
