# Supplementary material for: Transcriptome analysis of the differential effect of the NADPH oxidase gene RbohB in Phaseolus vulgaris roots following Rhizobium tropici and Rhizophagus irregularis inoculation
Source: BMC Genomics. 2019 Nov 4;20:800. doi: 10.1186/s12864-019-6162-7 (PMC6827182; doi:10.1186/s12864-019-6162-7)
Supplement: Supplementary file 5 — Additional file 2: Figure S2. Functional annotation of the DEGs in rhizobia-inoculated (Rhiz) and mycorrhized (Myc) roots of P. vulgaris. The bars show the percentages of the upregulated (a, c, e) and downregulated (b, d, f) DEGs annotated with GO terms for the functional categories of biological processes (a–b), molecular functions (c–d), and cellular components (e–f). A cutoff threshold of Log2FC ≥ 1.5 and P-adj/FDR ≤ 0.05 was used, and GO terms were assigned using a GO-Slim analysis of the UniProt database in Blast2GO. [file 12864_2019_6162_MOESM5_ESM.pdf]

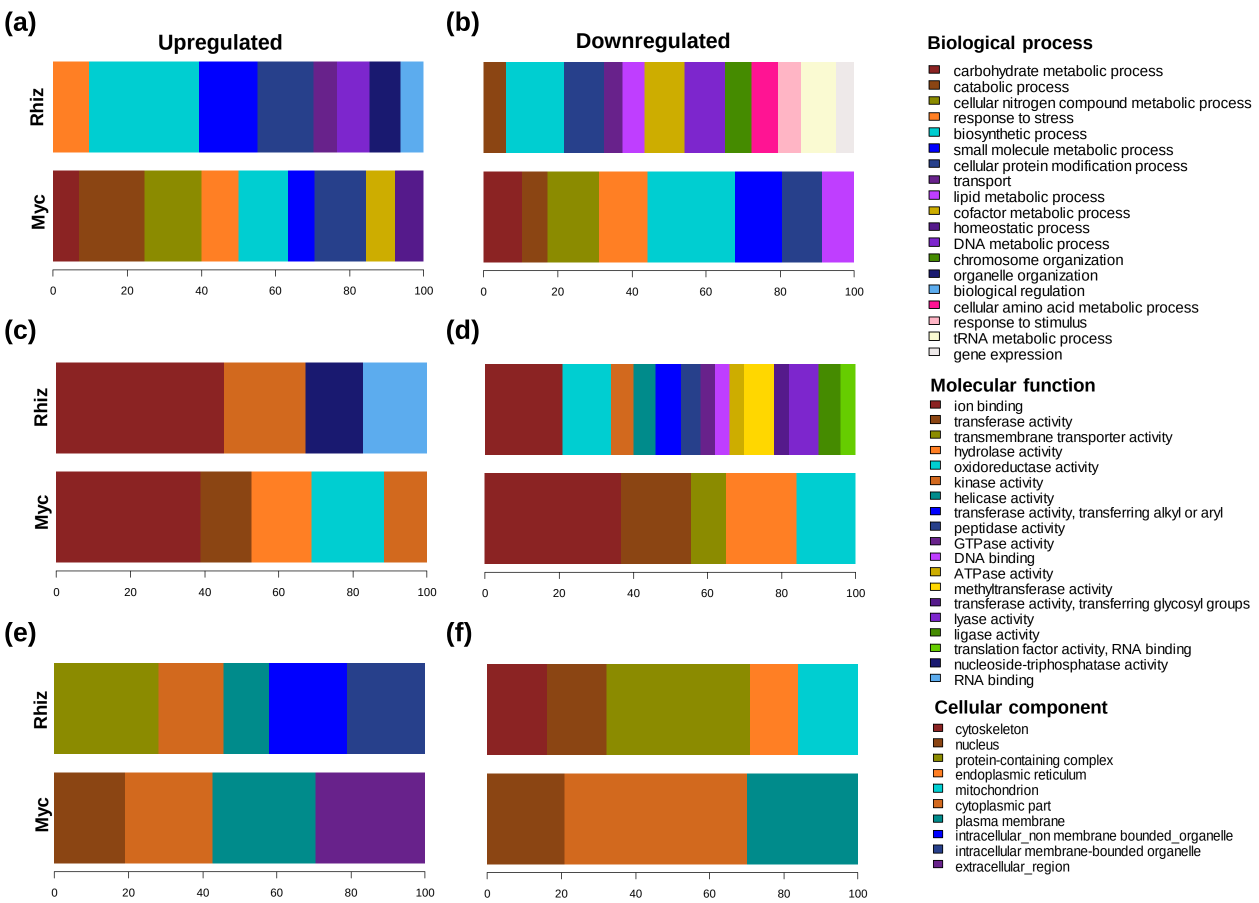

Figure S2 Functional annotation of the DEGs in rhizobia-inoculated (Rhiz) and mycorrhized (Myc) roots of *P. vulgaris*. The bars show the percentages of the upregulated (**a, c**, **e**) and downregulated (**b**, **d**, **f**) DEGs annotated with GO terms for the functional categories of biological processes (**a**–**b**), molecular functions (**c**–**d**), and cellular components (**e**–**f**). A cutoff threshold of Log2FC ≥ 1.5 and P-adj/FDR ≤ 0.05 was used, and GO terms were assigned using a GO-Slim analysis of the UniProt database in Blast2GO.
