# Supplementary material for: Transcriptome analysis of the differential effect of the NADPH oxidase gene RbohB in Phaseolus vulgaris roots following Rhizobium tropici and Rhizophagus irregularis inoculation
Source: BMC Genomics. 2019 Nov 4;20:800. doi: 10.1186/s12864-019-6162-7 (PMC6827182; doi:10.1186/s12864-019-6162-7)
Supplement: Supplementary file 6 — Additional file 6: Figure S3. Functional annotation of the DEGs in the PvRbohB-RNAi roots in nonsymbiotic conditions of P. vulgaris. Functional annotation of the upregulated and downregulated DEGs annotated with GO terms of the functional categories of biological processes (a), molecular functions (b), and cellular components (c). A cutoff threshold of Log2FC ≥ 1.5 and P-adj/FDR ≤ 0.05 was used, and GO terms were assigned using a GO-Slim analysis of the UniProt database in Blast2GO. [file 12864_2019_6162_MOESM6_ESM.pdf]

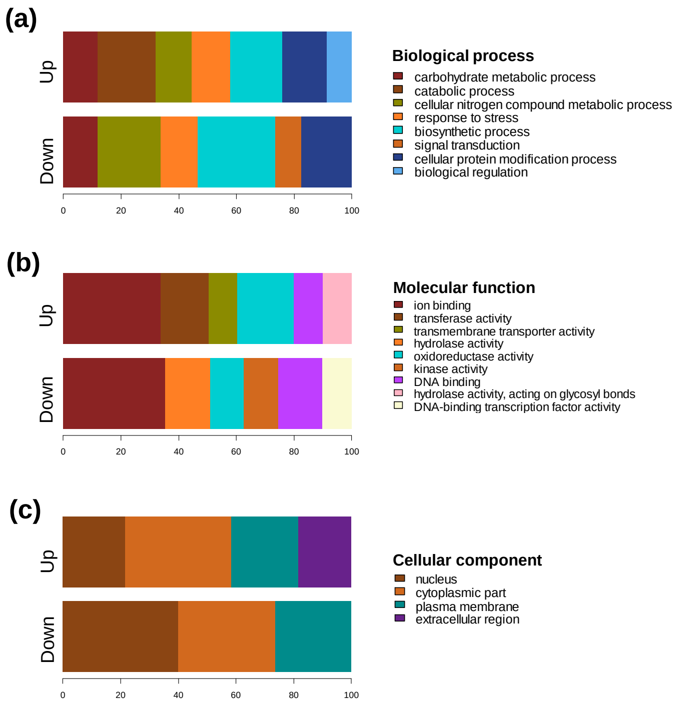

Figure S3 Functional annotation of the DEGs in the *PvRbohB*-RNAi roots in nonsymbiotic conditions of *P. vulgaris*. Functional annotation of the upregulated and downregulated DEGs annotated with GO terms of the functional categories of biological processes (**a**), molecular functions (**b**), and cellular components (**c**). A cutoff threshold of Log2FC $\geq$ 1.5 and P-adj/FDR $\leq$ 0.05 was used, and GO terms were assigned using a GO-Slim analysis of the UniProt database in Blast2GO.
