# Supplementary material for: Transcriptome analysis of the differential effect of the NADPH oxidase gene RbohB in Phaseolus vulgaris roots following Rhizobium tropici and Rhizophagus irregularis inoculation
Source: BMC Genomics. 2019 Nov 4;20:800. doi: 10.1186/s12864-019-6162-7 (PMC6827182; doi:10.1186/s12864-019-6162-7)
Supplement: Supplementary file 7 — Additional file 7: Figure S4. Functional annotation of the DEGs in rhizobia-inoculated and mycorrhized PvRbohB-RNAi roots, relative to the control. The bars show the percentages of upregulated (a, c, e) and downregulated (b, d, f) DEGs annotated with GO terms of the functional categories of biological processes (a–b), molecular functions (c–d), and cellular components (e–f). A cutoff threshold of Log2FC ≥ 1.5 and P-adj/FDR ≤ 0.05 was used, and GO terms were assigned using a GO-Slim analysis against the UniProt database in Blast2GO. [file 12864_2019_6162_MOESM7_ESM.pdf]

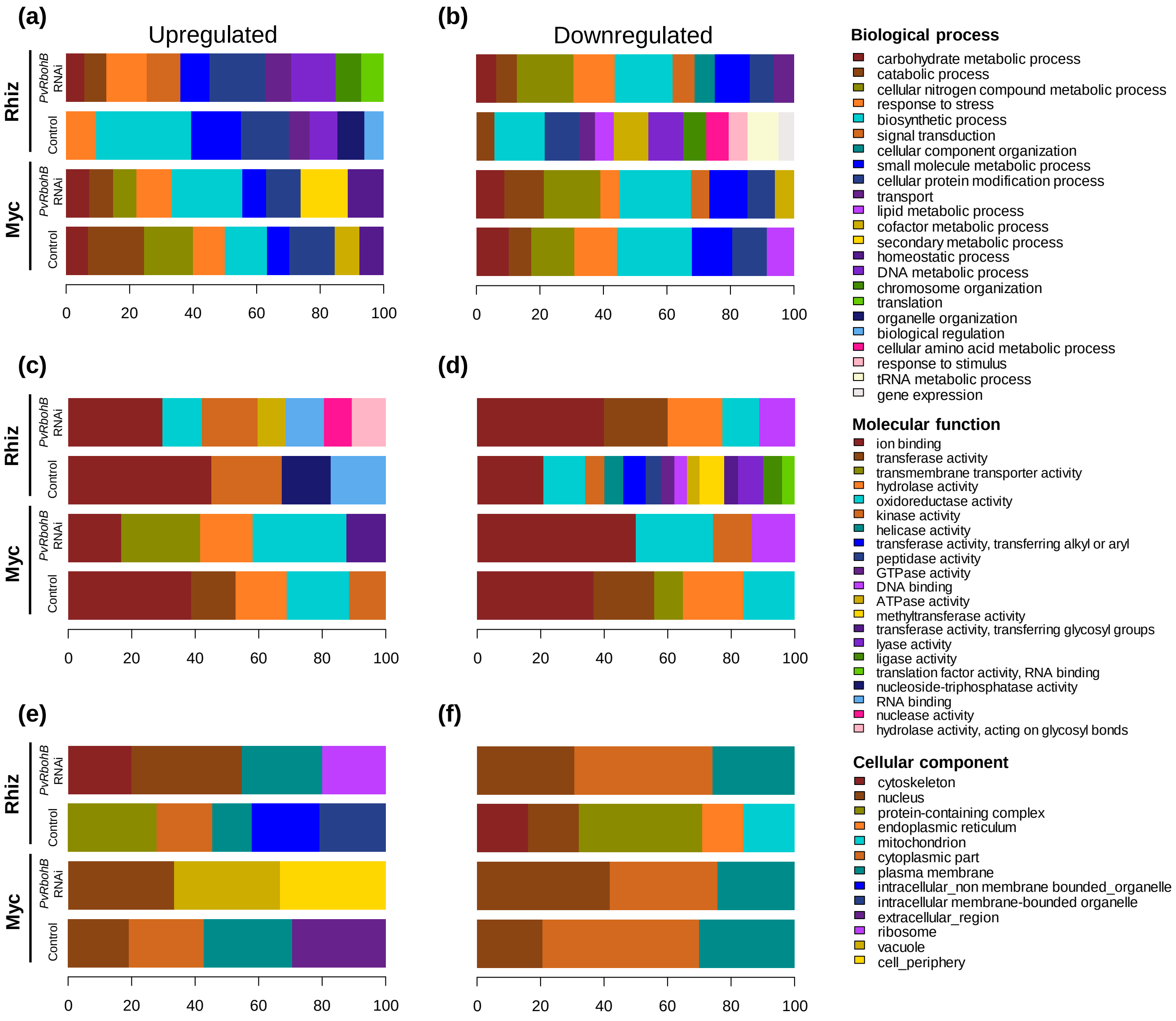


Figure S4 Functional annotation of the DEGs in rhizobia-inoculated and mycorrhized *PvRbohB*-RNAi roots, relative to the control. The bars show the percentages of upregulated (**a, c**, **e**) and downregulated (**b**, **d**, **f**) DEGs annotated with GO terms of the functional categories of biological processes (**a**–**b**), molecular functions (**c**–**d**), and cellular components (**e**–**f**). A cutoff threshold of Log2FC $\geq$ 1.5 and P-adj/FDR $\leq$ 0.05 was used, and GO terms were assigned using a GO-Slim analysis against the UniProt database in Blast2GO.
