# Supplementary material for: Transcriptome analysis of the differential effect of the NADPH oxidase gene RbohB in Phaseolus vulgaris roots following Rhizobium tropici and Rhizophagus irregularis inoculation
Source: BMC Genomics. 2019 Nov 4;20:800. doi: 10.1186/s12864-019-6162-7 (PMC6827182; doi:10.1186/s12864-019-6162-7)
Supplement: Supplementary file 8 — Additional file 8: Figure S5. Transcript levels of PvRbohB in the control and PvRbohB-RNAi roots used in this study. The plot includes RNA-Seq data and validating RT-qPCR data. Bars indicate the means ± SE. [file 12864_2019_6162_MOESM8_ESM.pdf]

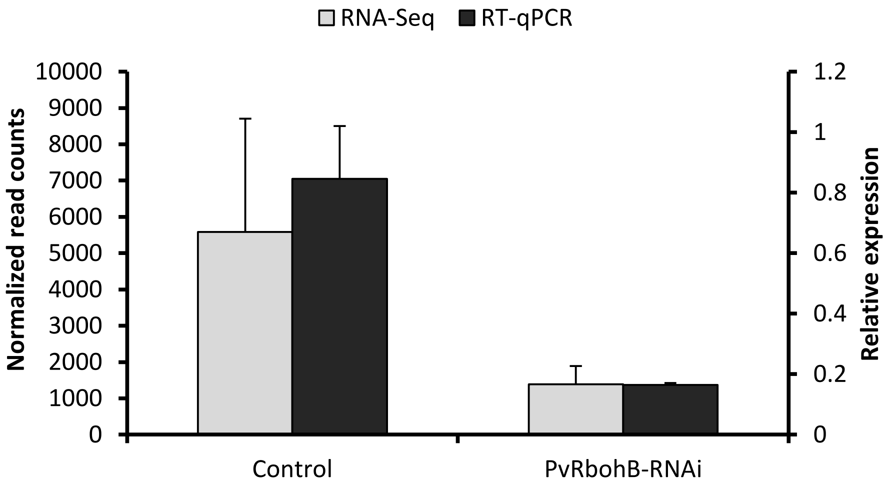

Figure S5 Transcript levels of *PvRbohB* in the control and *PvRbohB*-RNAi roots used in this study. The plot includes RNA-Seq data and validating RT-qPCR data. Bars indicate the means ± SE.
