# Supplementary material for: Transcriptome analysis of the differential effect of the NADPH oxidase gene RbohB in Phaseolus vulgaris roots following Rhizobium tropici and Rhizophagus irregularis inoculation
Source: BMC Genomics. 2019 Nov 4;20:800. doi: 10.1186/s12864-019-6162-7 (PMC6827182; doi:10.1186/s12864-019-6162-7)
Supplement: Supplementary file 15 — Additional file 15: Figure S12. KEGG pathway map of plant hormone signal transduction for upregulated genes in mycorrhized PvRbohB-silenced roots. [file 12864_2019_6162_MOESM15_ESM.pdf]

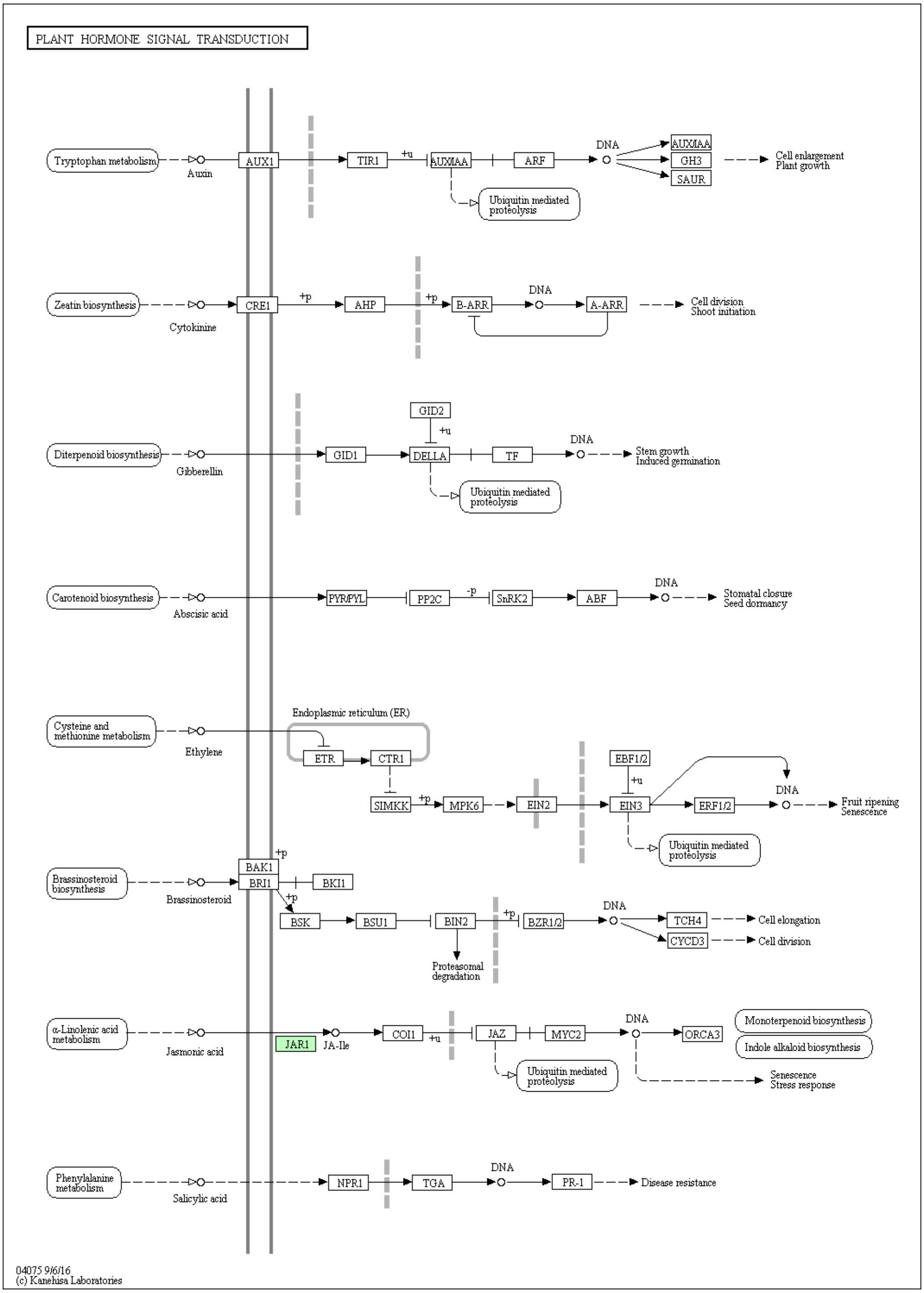

Figure S12 KEGG pathway map of plant hormone signal transduction for upregulated genes in mycorrhized *PvRbohB*-silenced roots.
