# Supplementary material for: Transcriptome analysis of the differential effect of the NADPH oxidase gene RbohB in Phaseolus vulgaris roots following Rhizobium tropici and Rhizophagus irregularis inoculation
Source: BMC Genomics. 2019 Nov 4;20:800. doi: 10.1186/s12864-019-6162-7 (PMC6827182; doi:10.1186/s12864-019-6162-7)
Supplement: Supplementary file 17 — Additional file 17: Figure S14. Correlation of the RNA-seq data and RT-qPCR expression profiles. The mean transcriptional changes (Log2FoldChange) of ROS-scavenging, cell wall, and phytohormone-related genes between the rhizobial-inoculated (a) and AM (b) roots at 7 dpi. [file 12864_2019_6162_MOESM17_ESM.pdf]

(a)

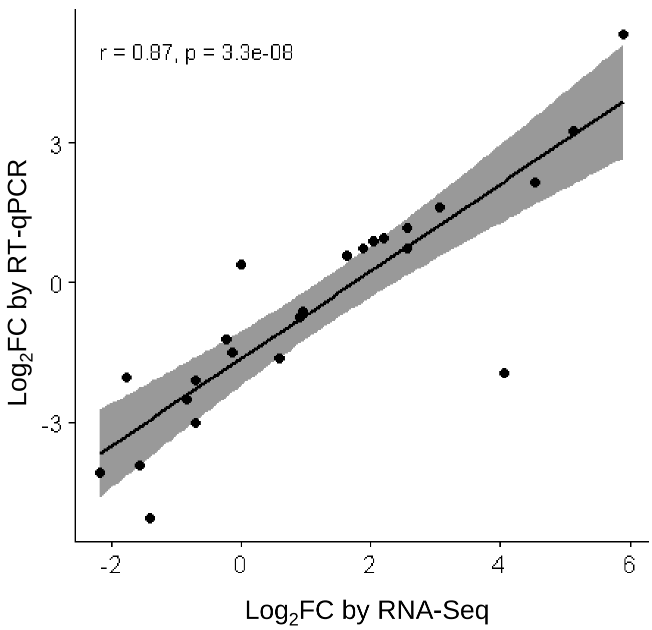

(b)

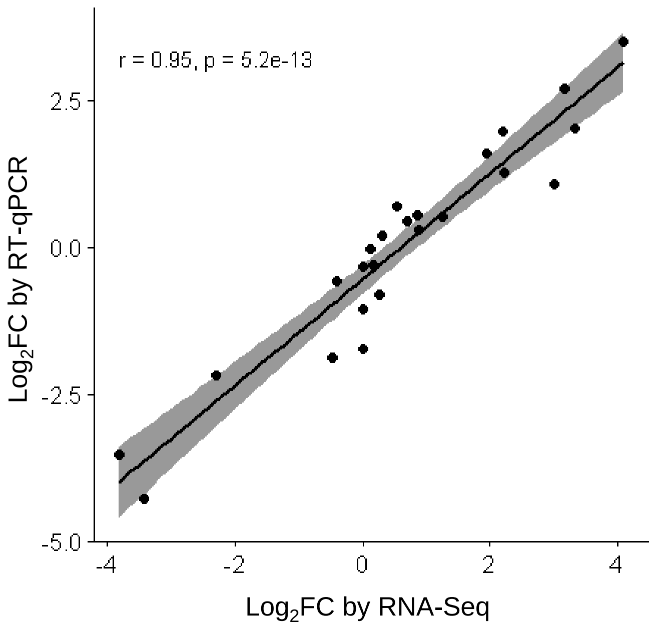

Figure S14 Correlation of the RNA-seq data and RT-qPCR expression profiles. The mean transcriptional changes (Log2FoldChange) of ROS-scavenging, cell wall, and phytohormone-related genes between the rhizobial-inoculated (**a**) and AM (**b**) roots at 7 dpi.
